# Time-dependent pattern of liver injury biomarkers in neonates with hypoxic-ischemic encephalopathy undergoing therapeutic hypothermia

**DOI:** 10.1007/s00431-026-07167-z

**Published:** 2026-06-15

**Authors:** Jannes Warlop, Noor Borloo, Hemananda Muniraman, Barbara Michniewicz, Kata Kovacs, Pieter Annaert, Gozdem Kayki, Paul Clarke, Dawid Szpecht, Miklos Szabo, Steffen Fieuws, Nadir Yalcin, Anne Smits, Karel Allegaert

**Affiliations:** 1 Neonatal Intensive Care Unit, University Hospitals Leuven, Louvain, Belgium; 2 Neonatal Intensive Care Unit, Norfolk and Norwich University Hospitals NHS Foundation Trust, Norwich, Norfolk, UK; 3 Neonatal Intensive Care Unit, Los Angeles General Medical Center, Los Angeles, USA; 4 Department of Neonatology, Poznan University of Medical Sciences, Poznan, Poland; 5 Department of Neonatology Pediatric Centre, Semmelweis University, Budapest, Hungary; 6 Drug Delivery and Disposition, Department of Pharmaceutical and Pharmacological Sciences, KU Leuven, Louvain, Belgium; 7 Division of Neonatology, Department of Pediatrics, Hacettepe University, Ankara, Türkiye; 8 Norwich Medical School, University of East Anglia, Norwich, UK; 9 Interuniversity Centre for Biostatistics and Statistical Bioinformatics, Department of Public Health, KU Leuven, Louvain, Belgium; 10 Department of Clinical Pharmacy, Faculty of Pharmacy, Hacettepe University, Ankara, Türkiye; 11 Department of Development and Regeneration, KU Leuven, Herestraat 49, Postbox 611, 3000 Louvain, Belgium; 12 Department of Pharmaceutical and Pharmacological Sciences, KU Leuven, Louvain, Belgium; 13 Department of Hospital Pharmacy, Erasmus MC, Rotterdam, The Netherlands

**Keywords:** Liver injury, Biomarkers, Therapeutic hypothermia, Perinatal asphyxia, Hypoxic-ischemic encephalopathy, Drug-induced liver injury, Newborn

## Abstract

Hypoxic-ischemic encephalopathy (HIE) often results in multi-organ damage. Liver injury following HIE is typically characterized by time-dependent altered patterns of alanine aminotransferase (ALT), aspartate aminotransferase (AST) or total bilirubin (TB) concentrations. This study aims to describe age-dependent patterns of liver injury biomarkers during and after therapeutic hypothermia (TH). Liver injury biomarkers (ALT, AST, TB) over the first 10 days of postnatal age (PNA) from six cohorts, including 428 neonates with moderate-to-severe HIE treated with TH were pooled. Statistical modelling with linear mixed models and quantile regression was applied to assess trends and correlations with HIE severity, gestational age, and birth weight. ALT and AST concentrations were highest on PNA day 1 (median [IQR]: 37.5 [18.75–85.50] U/L), and 154 [79–345 U/L], respectively) and declined thereafter. ALT and AST values were both associated with HIE severity (*p* < 0.001). The AST/ALT (De Ritis) ratio remained > 3.0 throughout PNA days 1–10.

*Conclusion*: We characterize age-dependent reference values of liver-injury biomarkers in HIE neonates treated with TH. Along with PNA, HIE severity has a strong association with liver biomarkers, while the De Ritis ratio reflects ischemic hepatic injury throughout PNA (day 1–10). These age-dependent reference values can be used to assess intra- and interpatient variability, or to explore other causes of hepatic toxicity like drug-induced liver injury, preferably following external validation.

**Table:** 

**What is Known:** *• Moderate to severe hypoxic-ischemic encephalopathy in neonates undergoing therapeutic hypothermia is often associated with multi-organ damage, including liver injury.* *• Except for acid/base parameters, laboratory values are inconsistently collected and reported in this specific population, so that reference values are warranted.* **What is New:** *• We report time-dependent reference values of liver injury biomarkers during and after therapeutic hypothermia.* *• These reference values can be used to assess intra- or interpatient variability and patterns in this specific population.*

**Supplementary Information:**

The online version contains supplementary material available at 10.1007/s00431-026-07167-z.

## Introduction

### Background

Perinatal asphyxia in neonates arises from critical deprivation of oxygen (hypoxia) and/or impaired systemic blood flow (ischemia) during peripartum. The body responds to this hypoxic state by redistributing blood flow to prioritize oxygen delivery to vital organs such as the brain, heart, and adrenal glands. This occurs often at the expense of other organs, such as the kidney, bone marrow, or liver. This compensatory mechanism can result in significant damage to these organs [1–4].

The primary clinical concern in perinatal asphyxia is hypoxic-ischemic encephalopathy (HIE), reflecting the neurological injury caused by prolonged or severe oxygen deprivation of the brain. The extent of injury to various other organs, including kidney or liver partly depends on HIE severity, as recently quantified for kidney function [5]. Hypoxic-ischemic liver injury is reported to occur in approximately 40–60% of neonates with HIE, with some studies suggesting rates as high as 80% [6]. This injury usually presents with temporary raised concentrations of alanine aminotransferase (ALT), and aspartate aminotransferase (AST), or bilirubin. The degree of these elevations and postnatal patterns also relates to the severity of the hypoxic insult, but remain poorly described [6, 7].

For neonates with moderate to severe HIE, whole-body therapeutic hypothermia (TH) is standard of care to prevent and limit neurological damage [8]. While effective with a number needed to treat of 7–9 to prevent one additional negative outcome (death, or adverse long term neurological outcomes), still a relevant portion of neonates with moderate to severe HIE do not survive or have adverse long-term neurological outcomes [8]. Consequently, clinical trials assessing the efficacy and safety of additional secondary interventions (e.g., erythropoietin, melatonin, allopurinol, cannabinoids) are ongoing or have been conducted to assess their potential add-on benefit on outcome [9, 10]. These pharmacological interventions may have a positive or negative impact on liver injury patterns, either protective, or related to drug-induced liver injury (DILI).

At present, reference values for liver enzymes in this population during and following TH remain poorly reported. These gaps in reference laboratory values are well recognized, since, aside from acid/base parameters, laboratory values were among the least consistently collected and reported data in this specific population [11]. These shortages are not limited to TH neonates, since published information on laboratory values for neonates is sparse, not systematic and incomplete [12].

### Objectives

This study aims to describe time-dependent patterns and provide age-dependent reference distributions for liver enzyme concentrations during and following TH in moderate to severe HIE newborns by pooling datasets.

## Materials and methods

### Ethics

The pooling effort was conducted in accordance with the Declaration of Helsinki, and was approved by the Ethics Committee Research UZ/KU Leuven (S-66227) covering both data collection for the Leuven unit, and the pooled dataset effort. Since the data were retrospectively collected, or were pooled based on previously published datasets, the requirement for informed written consent was waived.

### Study design

The HepaCool study pooled datasets on liver enzymes, based on retrospective collected data or as previously published. Based on a structured literature search (PubMed, March 2022, see Supplemental Material) on laboratory values in HIE neonates undergoing TH, potential datasets on liver enzymes were identified. Corresponding authors were invited to share and pool data for this specific HepaCool project. We focused on biomarker-specific patterns of liver injury in the first 10 days of postnatal age (PNA), to include the post TH recovery phase in neonates undergoing TH.

### Participants and variables

The pooled dataset was a priori restricted to birth weight (BW), gestational age (GA), post-natal age (PNA; days 1–10), HIE severity, survival status, and liver injury markers (ALT, AST, total, direct, and indirect bilirubin) to enable pooling and to respect the ethics approval.

The De Ritis (AST/ALT) ratio was calculated to further distinguish between hepatic ischemia and primary hepatocellular injury, since elevated De Ritis ratios are typically associated with ischemic liver damage, making this metric a useful biomarker to assess the extent and nature of liver injury [13]. Additional perinatal data and information on comorbidities were not collected, reflecting a pragmatic approach, while recognizing also the limitations.

### Data sources and bias

Data were extracted from retrospectively collected or previously published datasets and reflected routine clinical care in the contributing centers. Potential sources of bias included retrospective data collection, inter-center variability in laboratory measurements, and decreasing data availability at later postnatal ages.

### Statistical analysis

Data were reported by median and interquartile range (IQR), or mean and standard deviation (SD) for continuous variables. Categorical variables were reported by frequency and percentages. Spearman correlations (rho) were used to report associations on PNA day 1. Linear mixed models were used to assess the evolution of ALT, AST, and total bilirubin over PNA, with restricted cubic splines (four knots) incorporated to accommodate potential nonlinearity in the trends over time. The choice of the covariance structures (random intercept with serial correlation, random intercept with a random linear slope and serial correlation, autoregressive heterogeneous structures) was based on the Akaike Information Criterion.

Logarithmic transformations were applied to AST and ALT values to achieve a more symmetric distribution of residuals, while visualizations were presented after back-transformation to the original scale. The De Ritis ratio was also log-transformed since a ratio is an asymmetrical measure. From these models, percentiles for individual predictions at each PNA (e.g., the 90th percentile) were derived, and percentiles from quantile regression (50th and 90th) approach were also visualized, noting that quantile regression assumes that missing data occur completely at random. Interaction effects were included in the linear mixed model to verify if the evolution was dependent on the HIE severity. All statistical analyses were performed using SAS software version 9.4 (Cary, North Carolina, United States of America).

## Results

### Participants and descriptive data

We pooled data from six international cohorts, comprising a total of 428 neonates with moderate to severe HIE treated with TH [7, 14–18].

This dataset included 428 neonates diagnosed with HIE who received TH. Details on cohort distribution across participating centers are provided in the online supplement. Clinical characteristics of the included neonates are listed in Table 1*.* The median (IQR) GA was 39.9 (38.0–40.6) weeks, and BW of 3330 (3000–3776) grams. Ten neonates had a gestational age < 36 weeks, of whom 7 were born at 35 weeks and 3 at < 35 weeks. Thirty-six neonates underwent TH despite being reported (in retrospect) as having mild HIE. Data availability for liver biomarkers (ALT/AST/total bilirubin) varied across the postnatal period. We had limited access to data in later PNA, likely reflecting clinical monitoring practices with age (absolute numbers/day are provided in the tables). Among neonates with available data on HIE grading, those with severe HIE had a higher in-hospital mortality rate (18.1% versus 6.8%) compared to moderate HIE. For additional details on the cohort characteristics, we refer to the HepaCool statistical analysis document as provided in the supplemental material.

**Table 1:** Clinical characteristics of the 428 included neonates [7, 14–18]

Variable	Statistic	
Gestational age (weeks)	N	428
	Median	39.8
	IQR	(38; 40.6)
	Range	(33; 42.6)
Birth weight (grams)	N	428
	Median	3330
	IQR	(3000; 3776)
	Range	(1450; 5200)
Status		
Discharged alive	n (%)	337/392 (86%)
In-hospital death	n (%)	55/392 (14%)
Status missing	n	36/428
Grade HIE		
Mild	n (%)	36/349 (10%)
Moderate	n (%)	204/349 (58%)
Severe	n (%)	109/349 (32%)
Grade missing	n	79/428

### Laboratory findings

#### Alanine aminotransferase observations

**Fig. 1:**
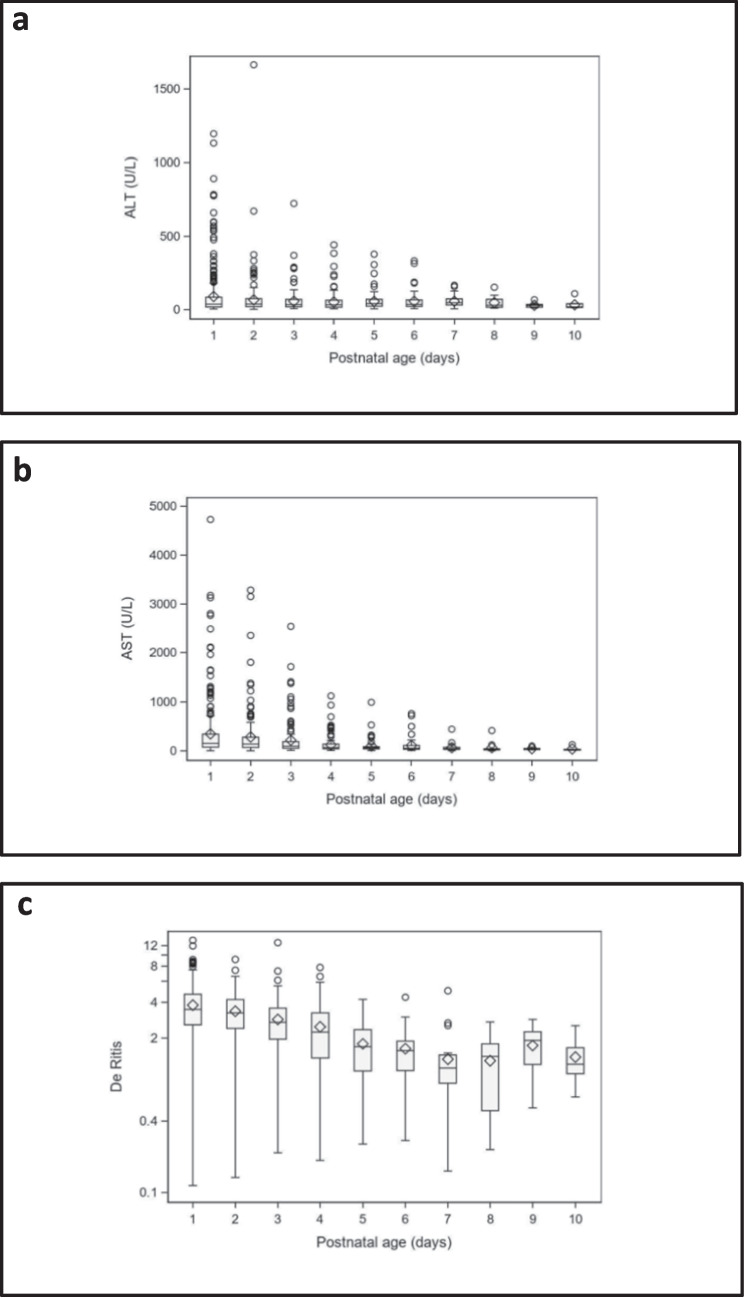
Trends and box plots for alanine aminotransferase (ALT) (**a**), aspartate aminotransferase (AST) (**b**), and De Ritis ratio (**c**) over postnatal age (PNA, day 1 to day 10)

**Fig. 2:**
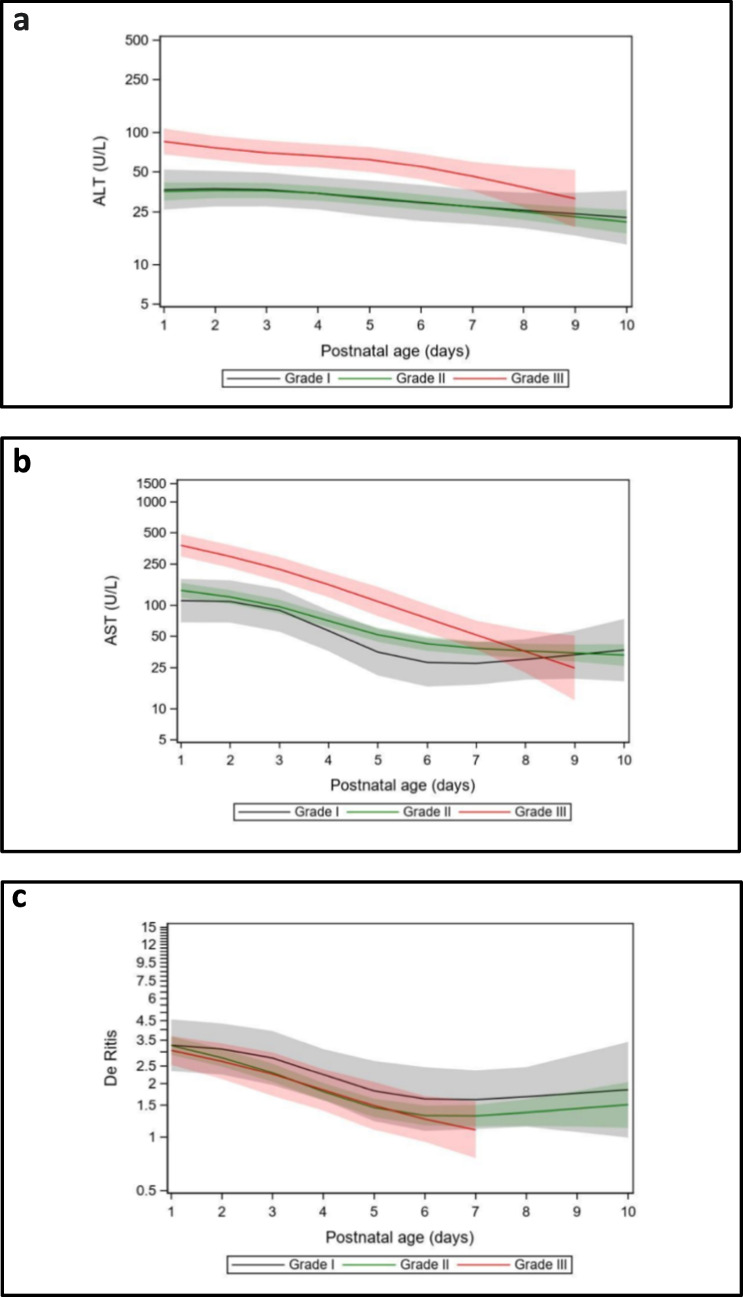
Estimated mean with 95% confidence interval for alanine aminotransferase (ALT, **a**), aspartate aminotransferase (AST, **b**), and De Ritis ratio over postnatal age (PNA, day 1 to day 10, **c**) in neonates with mild, moderate, or severe hypoxic-ischemic encephalopathy

Based on the pooled data, observed statistics (mean, SD, lower and upper quartile, minimum–maximum and 90th centile) over PNA days 1–10 from 1064 ALT (U/L) are provided in Table 2. ALT concentrations were markedly elevated at PNA day 1, with a median (IQR) of 37 (19–85) U/L, followed by a decline from PNA day 1 to day 3 (Fig. 1a). Estimated mean values with 95% confidence intervals (CI) at PNA day 1 were significantly higher (*p* < 0.001) in neonates with severe HIE, with a median (IQR) of 70 (30–186) versus 30 (17–64) U/L. (Fig. 2a). ALT on day 1 had a weak but significant correlation with GA (rho = 0.118, *p* = 0.03) and no significant correlation with BW (rho = 0.091, *p* = 0.10). Averaged over the PNA days, concentrations were significantly higher (*p* < 0.001) in neonates with severe HIE. However, the evolution of ALT was not significantly different between subjects with a different HIE grades (interaction effect, *p* = 0.39).

**Table 2:** Observed statistics (median, lower and higher quartile, minimum–maximum, and 90th centile) over postnatal age of 1064 alanine aminotransferase (ALT, U/L) observations, based on the pooled data

PNA (days)	N Obs	Median	Lower Quartile	Upper Quartile	Minimum	Maximum	90th
1	**324**	37	19	85	5	1195	191
2	**196**	38	21	75	3	1665	114
3	**174**	35	19	68	7	722	105
4	**142**	33	17	65	6	441	105
5	**96**	41	23	70	6	377	107
6	**57**	36	22	68	7	332	112
7	**47**	47	32	74	6	167	110
8	**12**	30	16	70	10	153	99
9	**9**	29	17	36	11	68	68
10	**7**	18	15	41	13	109	109

#### Aspartate aminotransferase observations

Observed statistics over days 1–10 PNA from 848 AST observations (U/L) are provided in Table 3. A similar trend is seen in AST concentrations, but with significantly higher absolute values compared to the ALT concentrations, with a median (IQR) of 154 (79–345) U/L, and a range from 5 to 4728 U/L (Fig. 1b). The association between AST concentrations and HIE severity was significant (*p* < 0.0001). Estimated means with 95% CI at PNA day 1 were significantly higher (*p* < 0.001) in neonates with severe HIE, with a median (IQR) of 405 (185–763) versus 124 (69–250) U/L (Fig. 2b). There was no significant correlation with either GA (rho = 0.05, *p* = 0.41) or BW (rho = 0.086, *p* = 0.16). While AST concentrations declined over PNA, the rate of decrease was slower in neonates with severe HIE, as indicated by a significant interaction effect (*p* = 0.01).

**Table 3:** Observed statistics (median, lower and higher quartile, minimum–maximum, and 90th centile) over postnatal age of 848 aspartate aminotransferase (AST, U/L) observations, based on the pooled data

PNA (days)	N Obs	Median	Lower Quartile	Upper Quartile	Minimum	Maximum	90th
1	**270**	154	79	345	5	4728	754
2	**154**	141	72	282	4	3282	586
3	**142**	96	55	191	14	2540	529
4	**112**	68	45	141	12	1128	295
5	**68**	64	43	88	9	997	273
6	**44**	54	34	112	9	763	223
7	**30**	43	31	75	7	446	128
8	**12**	28	21	44	5	417	125
9	**9**	35	27	51	21	103	103
10	**7**	26	18	33	18	129	129

De Ritis ratio observations. On PNA day 1, the median (IQR) De Ritis ratio was 3.47 (2.58–4.68), which is markedly elevated (Fig. 1c). Interestingly, averaged over the PNA days, the De Ritis ratio did not significantly differ between HIE severity grades (*p* = 0.53). Neither was the interaction effect between HIE grade and postnatal age for the De Ritis ratio significant (*p* = 0.60).

Total bilirubin concentrations are provided in Supplementary Table S1 and Supplementary Figure S1ab. Given the limited number of observations at later postnatal ages and the multiple physiological and clinical factors influencing bilirubin concentrations, these analyses were considered exploratory.

## Discussion

This multicenter retrospective study pooled data from six independent international cohorts comprising 428 neonates treated with TH. The primary objective was to characterize time-dependent patterns and its variability of liver biomarkers, specifically ALT, AST, and TB over the first 10 postnatal days. The analysis revealed distinct temporal patterns in liver enzyme concentrations, with an initial significant elevation in ALT and AST on day 1, with differences in the subsequent enzyme decline linked to the HIE severity, and a biphasic or fluctuating bilirubin pattern.

ALT concentrations were markedly elevated on PNA 1 and—although they declined thereafter—remained significantly higher in neonates with severe HIE (Fig. 1a and 2a). Interestingly, the trajectory of ALT decrease was parallel across HIE groups, suggesting that while the initial insult was more pronounced in severe cases, the overall recovery trajectory appeared broadly similar across HIE severity grades. In contrast, AST concentrations not only started at markedly high values but also declined at a slower rate in the severe HIE group (Fig. 1b and 2b). This prolonged AST elevation among the severe HIE neonates may indicate a sustained hepatic injury or leakage. Alternatively, raised AST concentrations can also relate to leakage from other tissues or organs, as AST is less specific for liver injury compared with ALT.

There are no widely accepted age-appropriate reference ranges for laboratory values in neonates against which our findings can be compared, while published information on laboratory values for neonates is sparse, not systematic, and incomplete, also in the TH population [11, 12]. However, the International Neonatal Consortium very recently launched a neonatal lab value open source tool to standardize lab reference ranges [19]. In Supplementary Table 2, we provide the extracted lab values from this tool for ALT, AST (median, IQR) for the first 10 days of postnatal age in term (37 weeks onwards) neonates. Compared to the similar lab values reported in Tables 2 and 3 in TH neonates, this comparison demonstrates a clear shift in the median and interquartile range, reflecting the liver injury in TH cases, and the relevance to report reference values in this specific population.

A noteworthy finding is the consistently elevated AST/ALT (De Ritis) ratio, which averaged 3.76 during the early postnatal period (Fig. 1c and 2c). Reflecting a mechanism related to liver injury in this population, elevated De Ritis ratios are typically associated with ischemic hepatic injury rather than primary hepatocellular damage. The lack of significant differences in the De Ritis ratio across HIE severity groups suggests that the underlying ischemic mechanism may be a common pathway in the pathophysiology of liver injury following perinatal asphyxia, despite variation in absolute enzyme concentrations. A change in this De Ritis ratio in a newborn undergoing TH may indicate an alternative mechanism of liver injury, such as drug-induced liver injury (DILI).

Total bilirubin data should be interpreted cautiously because bilirubin concentrations are influenced by multiple physiological and pathological factors, while the number of observations at later postnatal ages was limited (Supplemental Table 1 and Fig. 1ab). Therefore, these analyses should be considered exploratory, and cautious.

These findings support the notion that the liver is a vulnerable organ in the context of perinatal asphyxia. The compensatory redistribution of blood flow during hypoxia, intended to protect vital organs such as the brain and heart, may inadvertently compromise hepatic perfusion, resulting in ischemia and subsequent – most commonly—transient injury. In addition, the potential role of reactive oxygen species in exacerbating liver damage has been highlighted in both animal and in vitro studies, reinforcing the importance of oxidative stress in the cascade of hypoxic-ischemic injury [1–4].

Clinically, establishing reliable reference values for liver enzymes in neonates undergoing TH is crucial. Such benchmarks may facilitate risk stratification and the early identification of infants at potential risk for sustained hepatic dysfunction or DILI when additional pharmacological interventions are considered. Such interventions may relate to supportive therapy or clinical trials. Moreover, quantifying the extent and duration of hepatic injury provides insight into potential long-term outcomes and could inform future modifications to therapeutic protocols.

Despite its strengths, including the sizable, pooled standardized dataset and detailed temporal analysis of liver biomarkers, our study has several limitations. The retrospective design and inter-cohort variability in data collection methods or assays introduce potential selection bias and limit the generalizability of our findings. This includes also shortages related to likely relevant covariates that were not collected in the pooling effort, like Apgar score, the Score for Neonatal Acute Physiology – Perinatal Extension II (SNAPPE-II) score or other indicators of disease severity or likelihood of mortality. Furthermore, data beyond the first 7 postnatal days were sparse. This somewhat constrained our ability to fully characterize temporal trends. Finally, the absence of detailed perinatal information and comorbidity profiles limits the exploration of confounding factors that may influence liver enzyme concentrations.

## Conclusion

Our study provides a detailed overview of liver enzyme time-dependent dynamics in neonates with HIE who were treated with TH. The observed patterns underscore the liver’s susceptibility to hypoxic-ischemic injury, particularly in the context of severe HIE, and highlight the potential utility of biomarkers such as the De Ritis ratio in delineating the nature of hepatic damage. Future prospective studies with standardized data collection are warranted to confirm these findings and to further elucidate the implications for neonatal intensive care and long-term hepatic outcomes.

## Supplementary Information

Below is the link to the electronic supplementary material.

Supplementary file1 (DOCX 1131 KB)

Supplementary file2 (DOCX 182 KB)

## Data Availability

The pooled datasets were obtained for this analysis, and remain the property of the contributing groups, so that publicly sharing the individual data is not possible. Researchers interested in using the data can contact the corresponding author.

## Abbreviations

ALT: Alanine aminotransferase
AST: Aspartate aminotransferase
BW: Birth weight
DILI: Drug induced liver injury
GA: Gestational age
HIE: Hypoxic-ischemic encephalopathy
IQR: Interquartile range
PNA: Postnatal age (in days)
SD: Standard deviation
TB: Total bilirubin
TH: Therapeutic Hypothermia

## References

[1] Polglase GR, Ong T, Hillman NH (2016) Cardiovascular alterations and multiorgan dysfunction after birth asphyxia. Clin Perinatol 43:469–483. 10.1016/j.clp.2016.04.006 27524448 10.1016/j.clp.2016.04.006 PMC4988334

[2] Iribarren I, Hilario E, Álvarez A, Alonso-Alconada D (2022) Neonatal multiple organ failure after perinatal asphyxia. An Pediatr 97:e1-280. 10.1016/j.anpede.2022.08.010 10.1016/j.anpede.2022.08.010 36115781

[3] Jensen A, Garnier Y, Berger R (1999) Dynamics of fetal circulatory responses to hypoxia and asphyxia. Eur J Obstet Gynecol Reprod Biol 2115(98):155–172. 10.1016/s0301-2115(98)00325-x 10.1016/s0301-2115(98)00325-x 10428339

[4] Long M, Brandon DH (2007) Induced hypothermia for neonates with hypoxic-ischemic encephalopathy. J Obstet Gynecol Neonatal Nurs 36:293–298. 10.1111/j.1552-6909.2007.00150.x 17489937 10.1111/j.1552-6909.2007.00150.x

[5] Krzyzanski W, Wintermark P, Annaert P, Groenendaal FD, Sahin S, Öncel MY, Armangil KE, Battin MR, Gunn AJ, Frymoyer A, Chock VY-L, Keles E, Mekahli D, van den Anker J, Smits A, Allegaert K (2023) A population model of time-dependent changes in serum creatinine in (Near)term neonates with hypoxic-ischemic encephalopathy during and after therapeutic hypothermia. AAPS J 26:4. 10.1208/s12248-023-00851-0 38051395 10.1208/s12248-023-00851-0 PMC11177850

[6] Elsadek AE, FathyBarseem N, Suliman HA, Elshorbagy HH, Kamal NM, Talaat IM, Al-Shokary AH, Abdel Maksoud YH, Ibrahim AO, Attia AM, Abdelhalim WA, Abdelghani WE (2021) Hepatic injury in neonates with perinatal asphyxia. Glob Pediatr Health 8:2333794X20987781. 10.1177/2333794X20987781 33614837 10.1177/2333794X20987781 PMC7868451

[7] Michniewicz B, Szpecht D, Sowińska A, Sibiak R, Szymankiewicz M, Gadzinowski J (2020) Biomarkers in newborns with hypoxic-ischemic encephalopathy treated with therapeutic hypothermia. Childs Nerv Syst 36:2981–2988. 10.1007/s00381-020-04645-z 32367165 10.1007/s00381-020-04645-z PMC7649177

[8] Zanelli SA, Wusthoff CJ, Lucke AM, Kaufman DA, Committee on Fetus and Newborn, Section on Neurology (2026) Therapeutic hypothermia for neonatal hypoxic-ischemic encephalopathy: clinical report. Pediatrics 157:e2025073627. 10.1542/peds.2025-073627 41581784 10.1542/peds.2025-073627

[9] Victor S, Rocha-Ferreira E, Rahim A, Hagberg H, Edwards D (2022) New possibilities for neuroprotection in neonatal hypoxic-ischemic encephalopathy. Eur J Pediatr 181:875–887. 10.1007/s00431-021-04320-8 34820702 10.1007/s00431-021-04320-8 PMC8897336

[10] Molloy EJ, El-Dib M, Juul SE, Benders M, Gonzalez F, Bearer C, Wu YW, Robertson NJ, Hurley T, Branagan A, Michael Cotten C, Tan S, Laptook A, Austin T, Mohammad K, Rogers E, Luyt K, Bonifacio S, Soul JS, Gunn AJ, Newborn Brain Society Guidelines and Publications Committee (2023) Neuroprotective therapies in the NICU in term infants: present and future. Pediatr Res 93:1819–27. 10.1038/s41390-022-02295-2 36195634 10.1038/s41390-022-02295-2 PMC10070589

[11] Peeples ES, Mietzsch U, Molloy E, deVeber G, Mohammad K, Soul JS, Guez-Barber D, Pilon B, Chau V, Bonifacio S, Afifi J, Craig A, Wintermark P, Newborn Brain Society Guidelines and Publications Committee (2025) Data collection variability across neonatal hypoxic-ischemic encephalopathy registries. J Pediatr 279:114476, 114476. 10.1016/j.jpeds.2025.114476 10.1016/j.jpeds.2025.114476 PMC13384812 39863078

[12] Allegaert K, Hildebrand H, Singh K, Turner MA (2023) The publication quality of laboratory values in clinical studies in neonates. Pediatr Res 94:96–98. 10.1038/s41390-022-02385-1 36550353 10.1038/s41390-022-02385-1 PMC10356592

[13] Shaikh SM, Varma A, Kumar S, Acharya S, Patil R (2024) Navigating disease management: a comprehensive review of the de ritis ratio in clinical medicine. Cureus 16:e64447. 10.7759/cureus.64447 39139333 10.7759/cureus.64447 PMC11319523

[14] Muniraman H, Gardner D, Skinner J, Paweletz A, Vayalakkad A, Chee YH, Clifford C, Sanka S, Venkatesh V, Curley A, Victor S, Turner MA, Clarke P (2017) Biomarkers of hepatic injury and function in neonatal hypoxic ischemic encephalopathy and with therapeutic hypothermia. Eur J Pediatr 176:1295–1303. 10.1007/s00431-017-2956-2 28741035 10.1007/s00431-017-2956-2

[15] Róka A, Vásárhelyi B, Bodrogi E, Machay T, Szabó M (2007) Changes in laboratory parameters indicating cell necrosis and organ dysfunction in asphyxiated neonates on moderate systemic hypothermia. Acta Paediatr 96:1118–1121. 10.1111/j.1651-2227.2007.00361.x 17590199 10.1111/j.1651-2227.2007.00361.x

[16] Vander Elst Z, Stultjens T, Annaert P, Clarke P, Iglesias-Platas I, Agathos E, Kaykı G, Laenen A, Yalçın N, Smits A, Allegaert K (2025) Mathematical albumin function for neonates undergoing therapeutic hypothermia in comparison with control neonates. J Clin Pharmacol 65:923–932. 10.1002/jcph.70003 39930975 10.1002/jcph.70003 PMC12202195

[17] Chu WY, Annink KV, Nijstad AL, Maiwald CA, Schroth M, Bakkali LE, van Bel F, Benders MJ, van Weissenbruch MM, Hagen A, Franz AR, Dorlo TP, Allegaert K, Huitema AD, ALBINO Study Group (2022) Pharmacokinetic/pharmacodynamic modelling of allopurinol, its active metabolite oxypurinol, and biomarkers hypoxanthine, xanthine and uric acid in hypoxic-ischemic encephalopathy neonates. Clin Pharmacokinet 61:321–333. 10.1007/s40262-021-01068-0 34617261 10.1007/s40262-021-01068-0 PMC8813842

[18] Deferm N, Annink KV, Faelens R, Schroth M, Maiwald CA, Bakkali LE, van Bel F, Benders MJ, van Weissenbruch MM, Hagen A, Smits A, Annaert P, Franz AR, Allegaert K, ALBINO Study Group (2021) Glomerular filtration rate in asphyxiated neonates under therapeutic whole-body hypothermia, quantified by mannitol clearance. Clin Pharmacokinet 60:897–906. 10.1007/s40262-021-00991-6 33611729 10.1007/s40262-021-00991-6 PMC8249265

[19] Critical Path Institute, International Neonatal Consortium (2025) C-Path’s International Consortium lauches groundbreaking neonatal lab values GUI tool to standardize reference ranges in neonatal trials. https://c-path.org/c-paths-international-neonatal-consortium-launches-groundbreaking-neonatal-lab-values-gui-tool-to-standardize-reference-ranges-in-neonatal-trials/#:~:text=Neo-LV%20provides%20a%20publicly%20accessible%2C%20interactive%20interface%20for,evidence-based%20decision-making%20in%20both%20clinical%20and%20regulatory%20settings. Accessed 25 May 2026.

