# Supplementary material for: Time-dependent pattern of liver injury biomarkers in neonates with hypoxic-ischemic encephalopathy undergoing therapeutic hypothermia
Source: Eur J Pediatr. 2026 Jun 15;185(7):501. doi: 10.1007/s00431-026-07167-z (PMC13269461; doi:10.1007/s00431-026-07167-z)

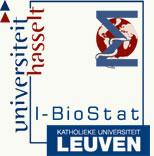


CHI

HepaCool

August 19, 2025

| *x* |
| --- |
| x |

Table of Contents

[***Info*** 3](#_Toc206508803)

[***1. Descriptives*** 4](#_Toc206508804)

[***Only subjects with grade HIE information*** 7](#_Toc206508805)

[***Correlations at Day 1*** 10](#_Toc206508806)

[***2. Total Bilirubine*** 11](#_Toc206508807)

[***2.1 Evolution all subjects*** 11](#_Toc206508808)

[***2.2 As a function of HIE grade*** 14](#_Toc206508809)

[***3. ALT*** 15](#_Toc206508810)

[***3.1 Evolution all subjects*** 15](#_Toc206508811)

[***3.2 As a function of HIE grade*** 19](#_Toc206508812)

[***4. AST*** 20](#_Toc206508813)

[***4.1 Evolution all subjects*** 20](#_Toc206508814)

[***4.2 As a function of HIE grade*** 24](#_Toc206508815)

[***5. De Ritis*** 25](#_Toc206508816)

[***5.1 Evolution all subjects*** 25](#_Toc206508817)

[***5.2 As a function of HIE grade*** 28](#_Toc206508818)

| ***Info*** |
| --- |

| Linear mixed models were used to model the evolution of total bilirubine, AST and ALT as a function of PNA. Restricted cubic splines (4 knots) were used to allow nonlinearity in the evolution. Various covariance structures were explored, the choice based on the AIC criterion: |
| --- |
| - random intercept and serial correlation |
| - random intercept, random linear slope and serial correlation |
| - autoregressive heterogeneous |
|  |
| A model with PNA as categorical variable (hence, not putting any structure on the evolution over time) was used as an alternative for the approach using splines. |
| A logaritmic transformation was applied for AST and ALT to obtain a more symmetric distribution of model residuals. De Ritis was also log-transformed since a ratio is an asymetrical measure. Visualisation was done after backtransformation to the original scale. |
| From these models, percentiles for individual predictions at each PNA can be derived (as an example, percentile 90 has been plotted). Percentiles 50 and 90 were also plotted from a quantile regression approach (note however that quantile regression assumes that missing values occur completely at random) |
|  |
| Interactions were added in the linear mixed model to verify if the evolution depends on the HIE grade. |
|  |
| All analyses have been performed using SAS software, version 9.4 of the SAS System for Windows. |
|  |
| Note that information becomes sparse as PNA increases. As a consequence results in later time period become unstable and need to be interpreted with caution. |

| ***1. Descriptives*** |
| --- |

| *Number of subjects* |
| --- |
| 428 |

| *Study* | *Frequency* |
| --- | --- |
| *RWD TH Cases Leuven* | 57 |
| *Hacettepe database* | 45 |
| *Muniraman databank* | 218 |
| *ROKA DATABANK* | 12 |
| *Michniewicz dataset* | 78 |
| *Nonmem dataset* | 18 |

| *Study* | *Last day with AST/ALT/Bili info* |
| --- | --- |
| RWD TH Cases Leuven | 10 |
| Hacettepe database | 9 |
| Muniraman databank | 7 |
| ROKA DATABANK | 4 |
| Michniewicz dataset | 1 |
| Nonmem dataset | 1 |

| *Variable* | *Statistic* | *All subjects* |
| --- | --- | --- |
| **STUDY** |  |  |
| RWD TH Cases Leuven | n/N (%) | 57/428 ( 13.32%) |
| Hacettepe database | n/N (%) | 45/428 ( 10.51%) |
| Muniraman databank | n/N (%) | 218/428 ( 50.93%) |
| ROKA DATABANK | n/N (%) | 12/428 ( 2.80%) |
| Michniewicz dataset | n/N (%) | 78/428 ( 18.22%) |
| Nonmem dataset | n/N (%) | 18/428 ( 4.21%) |
| **Gestational age (weeks)** | N | 428 |
|  | Mean | 39.24 |
|  | Std | 1.835 |
|  | Median | 39.86 |
|  | IQR | (38.00; 40.57) |
|  | Range | (33.00; 42.57) |
| **Birth weight (grams)** | N | 428 |
|  | Mean | 3399.14 |
|  | Std | 617.264 |
|  | Median | 3330.00 |
|  | IQR | (3000.00; 3776.50) |
|  | Range | (1450.00; 5200.00) |
| **Status** |  |  |
| Discharged alive | n/N (%) | 337/392 ( 85.97%) |
| In-hospital death | n/N (%) | 55/392 ( 14.03%) |
| **Grade HIE** |  |  |
| Grade I | n/N (%) | 36/349 ( 10.32%) |
| Grade II | n/N (%) | 204/349 ( 58.45%) |
| Grade III | n/N (%) | 109/349 ( 31.23%) |
| **Missing grade HIE** |  |  |
| HIE grade present | n/N (%) | 349/428 ( 81.54%) |
| HIE grade missing | n/N (%) | 79/428 ( 18.46%) |
|  | | |

| *Variable* | *Statistic* | *All subjects* |
| --- | --- | --- |
| **Last day with ALT/AST/Bili info** |  |  |
| 1 | n/N (%) | 104/428 ( 24.30%) |
| 2 | n/N (%) | 19/428 ( 4.44%) |
| 3 | n/N (%) | 29/428 ( 6.78%) |
| 4 | n/N (%) | 58/428 ( 13.55%) |
| 5 | n/N (%) | 51/428 ( 11.92%) |
| 6 | n/N (%) | 49/428 ( 11.45%) |
| 7 | n/N (%) | 80/428 ( 18.69%) |
| 8 | n/N (%) | 15/428 ( 3.50%) |
| 9 | n/N (%) | 13/428 ( 3.04%) |
| 10 | n/N (%) | 10/428 ( 2.34%) |
| **Number of days with ALT/AST/Bili info** |  |  |
| 1 | n/N (%) | 109/428 ( 25.47%) |
| 2 | n/N (%) | 28/428 ( 6.54%) |
| 3 | n/N (%) | 43/428 ( 10.05%) |
| 4 | n/N (%) | 79/428 ( 18.46%) |
| 5 | n/N (%) | 73/428 ( 17.06%) |
| 6 | n/N (%) | 50/428 ( 11.68%) |
| 7 | n/N (%) | 36/428 ( 8.41%) |
| 8 | n/N (%) | 6/428 ( 1.40%) |
| 9 | n/N (%) | 2/428 ( 0.47%) |
| 10 | n/N (%) | 2/428 ( 0.47%) |
|  | | |

| ***Only subjects with grade HIE information*** |
| --- |

| *Variable* | *Statistic* | *Grade I* | *Grade II* | *Grade III* | *P-value* |
| --- | --- | --- | --- | --- | --- |
| **STUDY** |  |  |  |  |  |
| RWD TH Cases Leuven | n/N (%) | 5/36 ( 13.89%) | 35/204 ( 17.16%) | 1/109 ( 0.92%) | <.001 |
| Muniraman databank | n/N (%) | 30/36 ( 83.33%) | 118/204 ( 57.84%) | 70/109 ( 64.22%) |  |
| ROKA DATABANK | n/N (%) | 1/36 ( 2.78%) | 10/204 ( 4.90%) | 1/109 ( 0.92%) |  |
| Michniewicz dataset | n/N (%) | 0/36 ( 0.00%) | 41/204 ( 20.10%) | 37/109 ( 33.94%) |  |
| **Gestational age (weeks)** | N | 36 | 204 | 109 | 0.067 |
|  | Mean | 40.00 | 39.24 | 39.31 |  |
|  | Std | 1.701 | 1.846 | 1.911 |  |
|  | Median | 40.07 | 39.72 | 40.00 |  |
|  | IQR | (39.22; 41.29) | (38.00; 40.65) | (38.00; 40.86) |  |
|  | Range | (36.00; 42.29) | (33.00; 42.57) | (35.00; 42.14) |  |
| **Birth weight (grams)** | N | 36 | 204 | 109 | 0.029 |
|  | Mean | 3673.75 | 3388.12 | 3421.80 |  |
|  | Std | 612.868 | 634.113 | 669.376 |  |
|  | Median | 3685.00 | 3345.00 | 3320.00 |  |
|  | IQR | (3295.00; 4178.00) | (2907.00; 3792.50) | (3000.00; 3840.00) |  |
|  | Range | (2250.00; 4620.00) | (1985.00; 5200.00) | (1450.00; 5160.00) |  |
| **Status** |  |  |  |  |  |
| Discharged alive | n/N (%) | 26/34 ( 76.47%) | 179/192 ( 93.23%) | 86/105 ( 81.90%) | 0.002 |
| In-hospital death | n/N (%) | 8/34 ( 23.53%) | 13/192 ( 6.77%) | 19/105 ( 18.10%) |  |
| **Last day with ALT/AST/Bili info** | N | 36 | 204 | 109 | 0.003 |
|  | Mean | 5.31 | 4.70 | 3.75 |  |
|  | Std | 1.910 | 2.551 | 2.561 |  |
|  | Median | 5.50 | 5.00 | 4.00 |  |
|  | IQR | (4.00; 7.00) | (3.00; 7.00) | (1.00; 7.00) |  |
|  | Range | (1.00; 10.00) | (1.00; 10.00) | (1.00; 7.00) |  |
| **Number of days with ALT/AST/Bili info** | N | 36 | 204 | 109 | 0.024 |
|  | Mean | 4.22 | 3.93 | 3.28 |  |
|  | Std | 1.396 | 2.050 | 2.241 |  |
|  | Median | 4.00 | 4.00 | 3.00 |  |
|  | IQR | (3.00; 5.00) | (2.00; 5.00) | (1.00; 5.00) |  |
|  | Range | (1.00; 7.00) | (1.00; 10.00) | (1.00; 7.00) |  |
| Variables presented with percentages are analysed using a Chi-square test. Variables summarized by means, medians,... are analysed using a Kruskal-Wallis test. All reported p-values are two-sided | | | | | |

| *Variable* | *Statistic* | *Grade I* | *Grade II* | *Grade III* |
| --- | --- | --- | --- | --- |
| **Last day with ALT/AST/Bili info** |  |  |  |  |
| 1 | n/N (%) | 1/36 ( 2.78%) | 42/204 ( 20.59%) | 42/109 ( 38.53%) |
| 2 | n/N (%) | 1/36 ( 2.78%) | 7/204 ( 3.43%) | 6/109 ( 5.50%) |
| 3 | n/N (%) | 4/36 ( 11.11%) | 14/204 ( 6.86%) | 4/109 ( 3.67%) |
| 4 | n/N (%) | 8/36 ( 22.22%) | 31/204 ( 15.20%) | 10/109 ( 9.17%) |
| 5 | n/N (%) | 4/36 ( 11.11%) | 25/204 ( 12.25%) | 8/109 ( 7.34%) |
| 6 | n/N (%) | 7/36 ( 19.44%) | 26/204 ( 12.75%) | 10/109 ( 9.17%) |
| 7 | n/N (%) | 8/36 ( 22.22%) | 36/204 ( 17.65%) | 29/109 ( 26.61%) |
| 8 | n/N (%) | 2/36 ( 5.56%) | 8/204 ( 3.92%) | 0/109 ( 0.00%) |
| 9 | n/N (%) | 0/36 ( 0.00%) | 11/204 ( 5.39%) | 0/109 ( 0.00%) |
| 10 | n/N (%) | 1/36 ( 2.78%) | 4/204 ( 1.96%) | 0/109 ( 0.00%) |
| **Number of days with ALT/AST/Bili info** |  |  |  |  |
| 1 | n/N (%) | 1/36 ( 2.78%) | 44/204 ( 21.57%) | 45/109 ( 41.28%) |
| 2 | n/N (%) | 2/36 ( 5.56%) | 11/204 ( 5.39%) | 6/109 ( 5.50%) |
| 3 | n/N (%) | 8/36 ( 22.22%) | 22/204 ( 10.78%) | 4/109 ( 3.67%) |
| 4 | n/N (%) | 11/36 ( 30.56%) | 42/204 ( 20.59%) | 16/109 ( 14.68%) |
| 5 | n/N (%) | 7/36 ( 19.44%) | 37/204 ( 18.14%) | 14/109 ( 12.84%) |
| 6 | n/N (%) | 5/36 ( 13.89%) | 25/204 ( 12.25%) | 13/109 ( 11.93%) |
| 7 | n/N (%) | 2/36 ( 5.56%) | 19/204 ( 9.31%) | 11/109 ( 10.09%) |
| 8 | n/N (%) | 0/36 ( 0.00%) | 3/204 ( 1.47%) | 0/109 ( 0.00%) |
| 10 | n/N (%) | 0/36 ( 0.00%) | 1/204 ( 0.49%) | 0/109 ( 0.00%) |
|  | | | | |

***Relation grade HIE and AST/ALT/Bilirubine at day 1***

| *Variable* | *Statistic* | *Grade I* | *Grade II* | *Grade III* | *P-value* |
| --- | --- | --- | --- | --- | --- |
| **Total bilirubin (mg/dL)** | N | 29 | 132 | 52 | 0.334 |
|  | Mean | 2.91 | 3.14 | 2.67 |  |
|  | Std | 1.504 | 1.564 | 1.260 |  |
|  | Median | 2.32 | 3.01 | 2.76 |  |
|  | IQR | (1.91; 4.00) | (1.86; 3.94) | (1.68; 3.39) |  |
|  | Range | (0.65; 6.96) | (0.81; 7.37) | (0.58; 6.21) |  |
| **AST (U/L)** | N | 17 | 130 | 65 | <.001 |
|  | Mean | 152.82 | 286.76 | 656.23 |  |
|  | Std | 118.155 | 577.762 | 707.038 |  |
|  | Median | 128.00 | 124.40 | 404.70 |  |
|  | IQR | (52.00; 209.00) | (69.00; 250.00) | (185.10; 763.40) |  |
|  | Range | (37.00; 401.00) | (5.00; 4728.00) | (43.00; 3126.00) |  |
| **ALT (U/L)** | N | 31 | 145 | 72 | <.001 |
|  | Mean | 44.65 | 64.89 | 172.44 |  |
|  | Std | 37.161 | 130.567 | 239.413 |  |
|  | Median | 27.00 | 30.00 | 70.50 |  |
|  | IQR | (20.00; 59.00) | (16.60; 64.00) | (30.00; 185.65) |  |
|  | Range | (10.00; 169.00) | (5.00; 1131.40) | (7.00; 1195.30) |  |
| **De Ritis** | N | 16 | 112 | 51 | 0.925 |
|  | Mean | 3.54 | 3.82 | 3.73 |  |
|  | Std | 1.456 | 2.096 | 1.964 |  |
|  | Median | 3.31 | 3.42 | 3.26 |  |
|  | IQR | (2.41; 4.44) | (2.52; 4.80) | (2.27; 4.89) |  |
|  | Range | (1.72; 7.30) | (0.16; 13.31) | (0.11; 8.81) |  |
| Variables presented with percentages are analysed using a Chi-square test. Variables summarized by means, medians,... are analysed using a Kruskal-Wallis test. All reported p-values are two-sided | | | | | |

| ***Correlations at Day 1*** |
| --- |

|  | *Spearman (95%CI)* | |  | | |
| --- | --- | --- | --- | --- | --- |
|  | | | | *P value* |  |
| Total bilirubin (mg/dL) | 0.147 | (0.027;0.261) |  | 0.0158 |  |
| AST (U/L) | 0.050 | (-0.070;0.169) |  | 0.4100 |  |
| ALT (U/L) | 0.118 | (0.009;0.224) |  | 0.0331 |  |
| De Ritis | -0.250 | (-0.365;-0.126) |  | <.0001 |  |
| Correlations at PNA=1 Overview Spearman correlations with Gestational age (weeks) P-value: raw p-value Spearman Correlation, based on Fishers Z transformation | | | | | |

|  | *Spearman (95%CI)* | |  | | |
| --- | --- | --- | --- | --- | --- |
|  | | | | *P value* |  |
| Total bilirubin (mg/dL) | 0.043 | (-0.077;0.161) |  | 0.4855 |  |
| AST (U/L) | 0.086 | (-0.034;0.203) |  | 0.1587 |  |
| ALT (U/L) | 0.091 | (-0.019;0.197) |  | 0.1039 |  |
| De Ritis | -0.152 | (-0.274;-0.025) |  | 0.0190 |  |
| Correlations at PNA=1 Overview Spearman correlations with Birth weight (grams) P-value: raw p-value Spearman Correlation, based on Fishers Z transformation | | | | | |

| ***2. Total Bilirubine*** |
| --- |

| ***2.1 Evolution all subjects*** |
| --- |

| ***Individual profiles*** |
| --- |


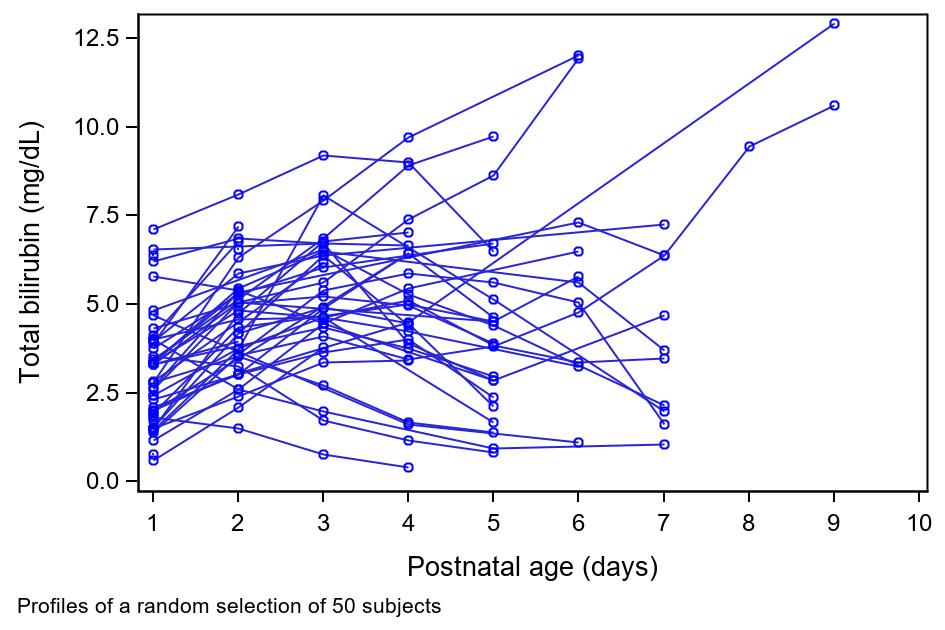


| ***Observed information*** |
| --- |

| *Analysis Variable : bilitot Total bilirubin (mg/dL)* | | | | | | | | | | |
| --- | --- | --- | --- | --- | --- | --- | --- | --- | --- | --- |
| *Postnatal age (days)* | *N Obs* | *Mean* | *Std Dev* | *Median* | *Lower Quartile* | *Upper Quartile* | *Minimum* | *Maximum* | *90th Pctl* | *95th Pctl* |
| 1 | 270 | 2.89 | 1.43 | 2.67 | 1.80 | 3.65 | 0.58 | 7.37 | 4.81 | 5.86 |
| 2 | 247 | 3.89 | 1.71 | 3.89 | 2.50 | 5.05 | 0.06 | 8.10 | 6.26 | 6.79 |
| 3 | 218 | 4.53 | 2.07 | 4.52 | 3.07 | 6.16 | 0.39 | 10.79 | 6.84 | 7.57 |
| 4 | 186 | 4.48 | 2.52 | 4.46 | 2.49 | 6.34 | 0.36 | 11.29 | 7.37 | 9.00 |
| 5 | 145 | 4.02 | 2.67 | 3.60 | 1.86 | 5.34 | 0.38 | 12.59 | 8.00 | 8.98 |
| 6 | 105 | 3.96 | 3.41 | 3.36 | 1.39 | 5.45 | 0.06 | 19.88 | 7.31 | 11.90 |
| 7 | 74 | 3.70 | 3.09 | 3.01 | 1.62 | 4.93 | 0.46 | 15.73 | 6.94 | 10.14 |
| 8 | 22 | 6.18 | 4.64 | 4.95 | 2.10 | 9.52 | 0.60 | 16.48 | 12.11 | 14.89 |
| 9 | 15 | 4.29 | 4.30 | 2.93 | 0.86 | 7.38 | 0.40 | 12.92 | 11.41 | 12.92 |
| 10 | 9 | 1.97 | 1.05 | 2.22 | 0.99 | 2.66 | 0.80 | 3.47 | 3.47 | 3.47 |


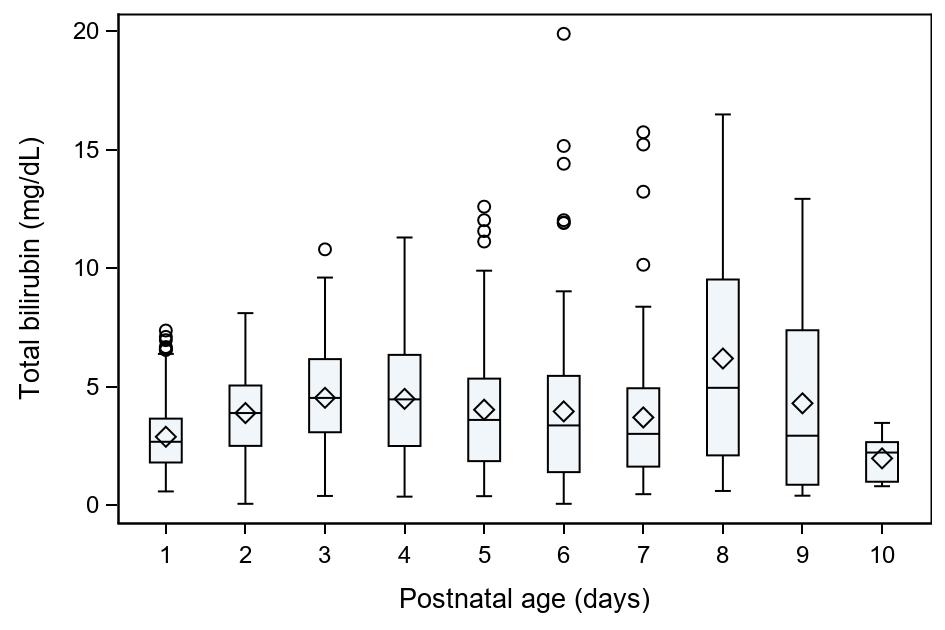


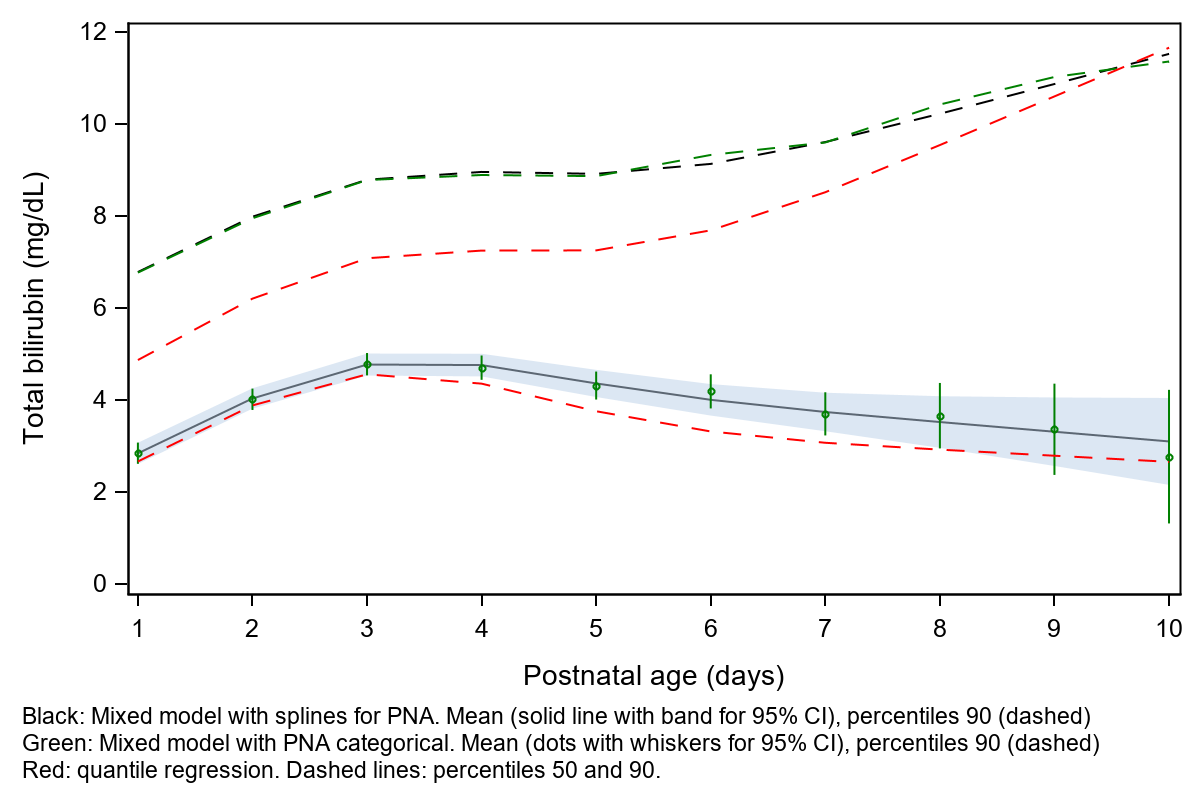


| ***2.2 As a function of HIE grade*** |
| --- |

| *label* | *Num DF* | *Den DF* | *F Value* | *P-value* |
| --- | --- | --- | --- | --- |
| Main effect HIE grade? | 2 | 558 | 2.16 | 0.1158 |
| Interaction with HIE grade? | 6 | 553 | 0.83 | 0.5474 |


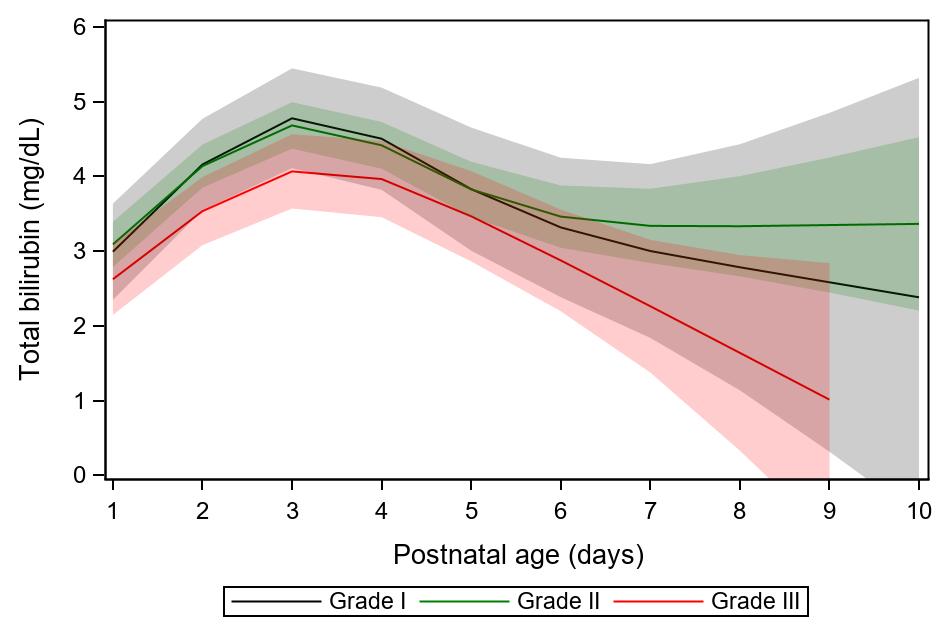


| ***3. ALT*** |
| --- |

| ***3.1 Evolution all subjects*** |
| --- |

| ***Individual profiles*** |
| --- |


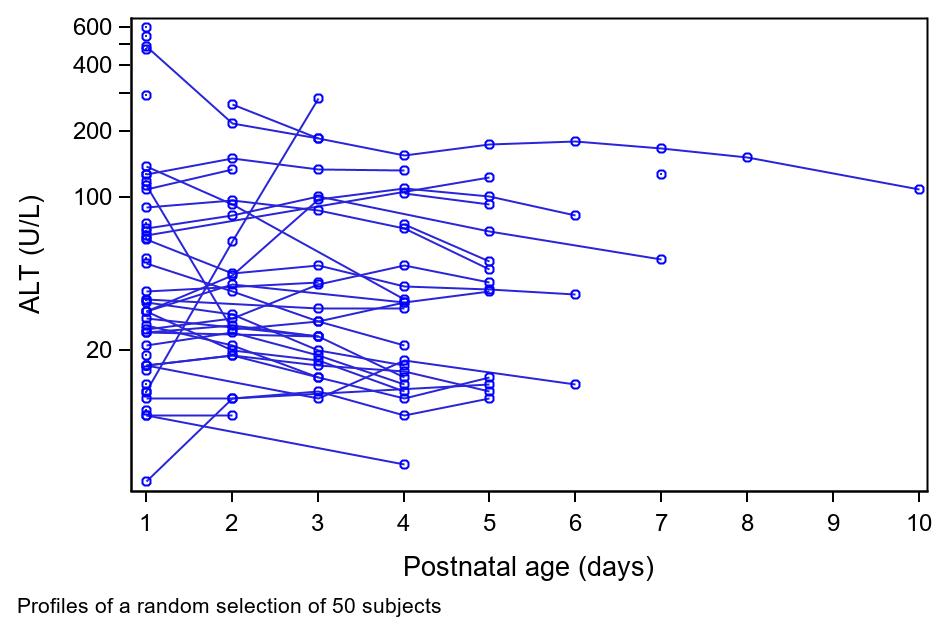


| ***Observed information*** |
| --- |

| *Analysis Variable : ALT ALT (U/L)* | | | | | | | | | | |
| --- | --- | --- | --- | --- | --- | --- | --- | --- | --- | --- |
| *Postnatal age (days)* | *N Obs* | *Mean* | *Std Dev* | *Median* | *Lower Quartile* | *Upper Quartile* | *Minimum* | *Maximum* | *90th Pctl* | *95th Pctl* |
| 1 | 324 | 89.81 | 158.34 | 37.50 | 18.75 | 85.50 | 5.00 | 1195.30 | 191.00 | 380.00 |
| 2 | 196 | 67.18 | 135.63 | 38.00 | 21.00 | 75.50 | 3.00 | 1665.00 | 114.00 | 219.00 |
| 3 | 174 | 57.76 | 74.59 | 35.00 | 20.00 | 70.00 | 7.00 | 722.00 | 106.00 | 187.00 |
| 4 | 142 | 52.30 | 62.35 | 33.00 | 17.00 | 65.00 | 6.00 | 441.00 | 105.00 | 133.00 |
| 5 | 96 | 56.44 | 57.24 | 41.00 | 23.50 | 70.50 | 6.00 | 377.00 | 107.00 | 158.00 |
| 6 | 57 | 57.25 | 64.05 | 36.00 | 22.00 | 68.00 | 7.00 | 332.00 | 112.00 | 188.00 |
| 7 | 47 | 57.23 | 36.98 | 47.00 | 32.00 | 74.00 | 6.00 | 167.00 | 110.00 | 127.00 |
| 8 | 12 | 48.17 | 43.41 | 30.00 | 16.50 | 70.50 | 10.00 | 153.00 | 99.00 | 153.00 |
| 9 | 9 | 30.22 | 17.61 | 29.00 | 17.00 | 36.00 | 11.00 | 68.00 | 68.00 | 68.00 |
| 10 | 7 | 33.43 | 34.60 | 18.00 | 15.00 | 41.00 | 13.00 | 109.00 | 109.00 | 109.00 |


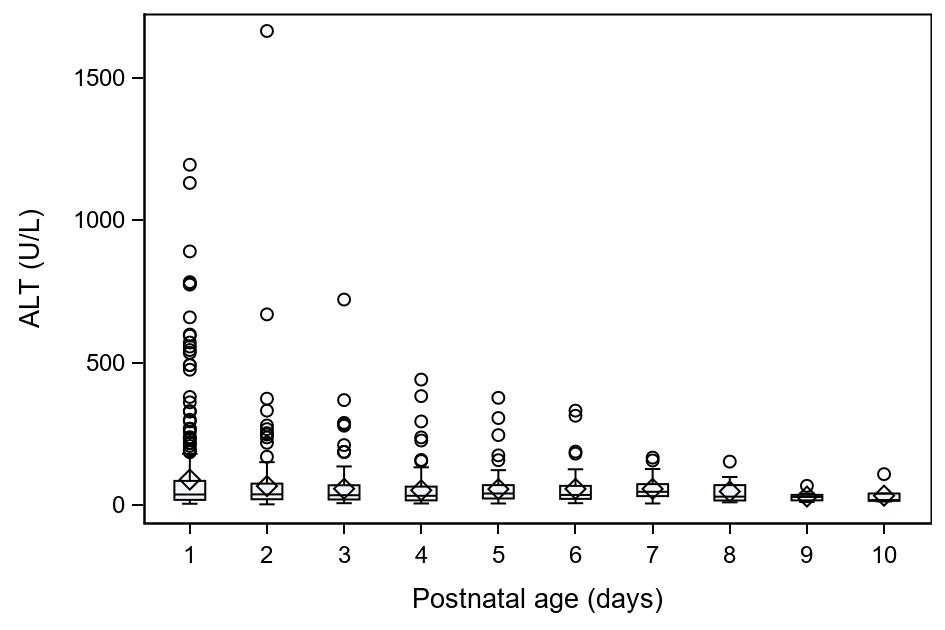


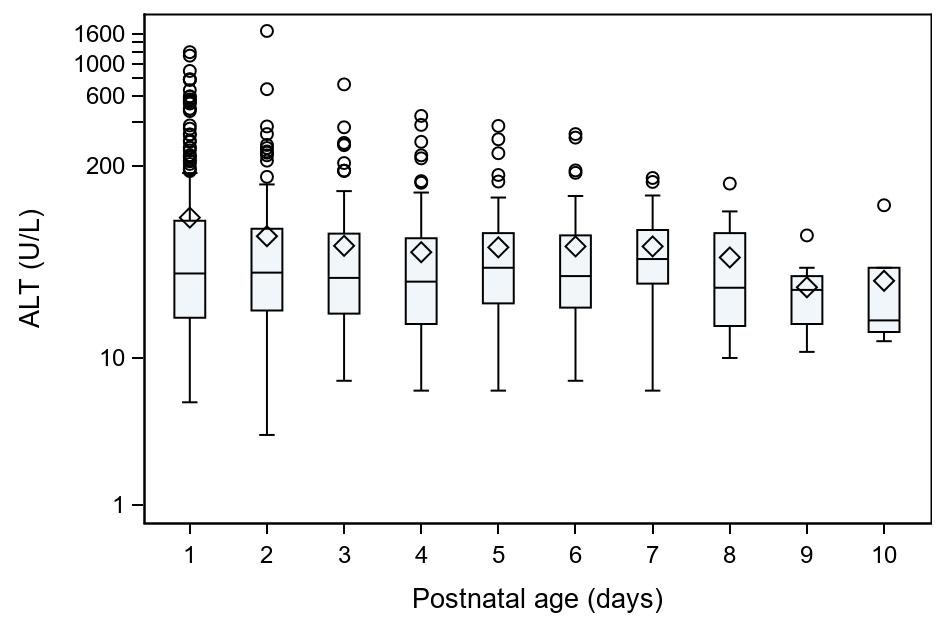


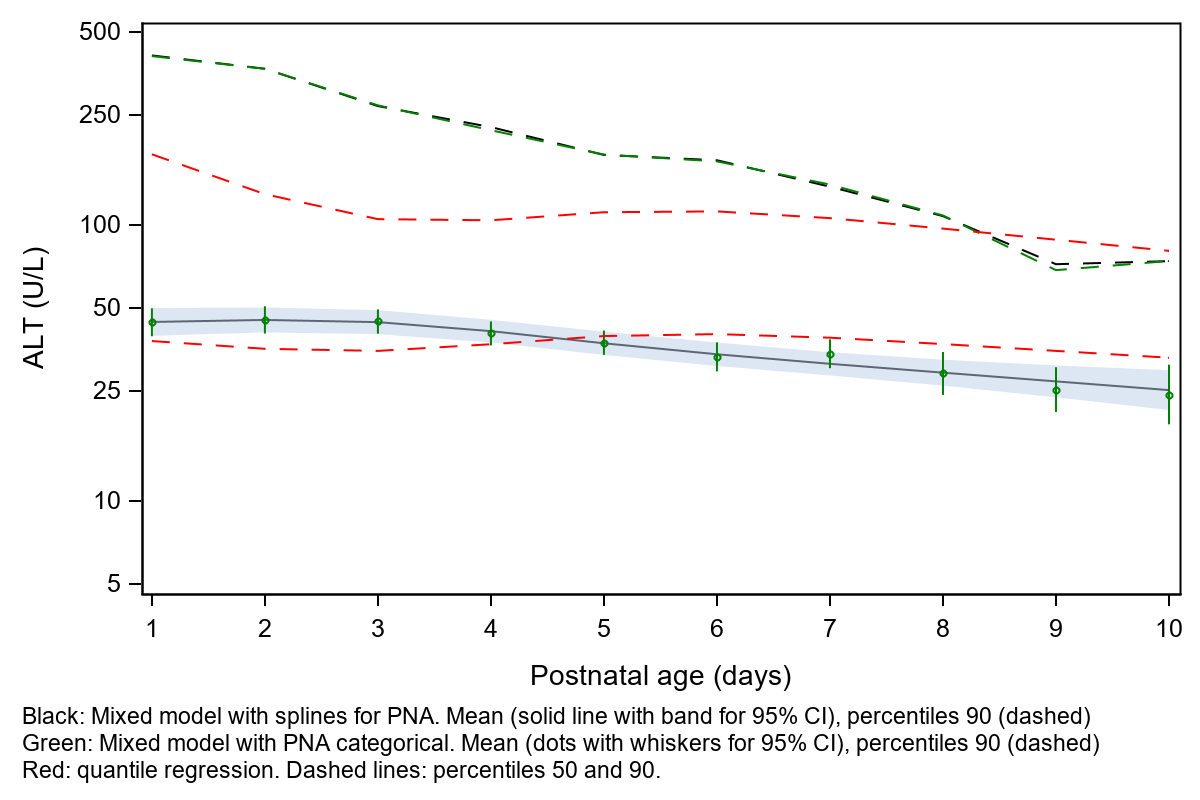


| ***3.2 As a function of HIE grade*** |
| --- |

| *label* | *Num DF* | *Den DF* | *F Value* | *P-value* |
| --- | --- | --- | --- | --- |
| Main effect HIE grade? | 2 | 294 | 20.52 | <.0001 |
| Interaction with HIE grade? | 6 | 491 | 1.04 | 0.3960 |


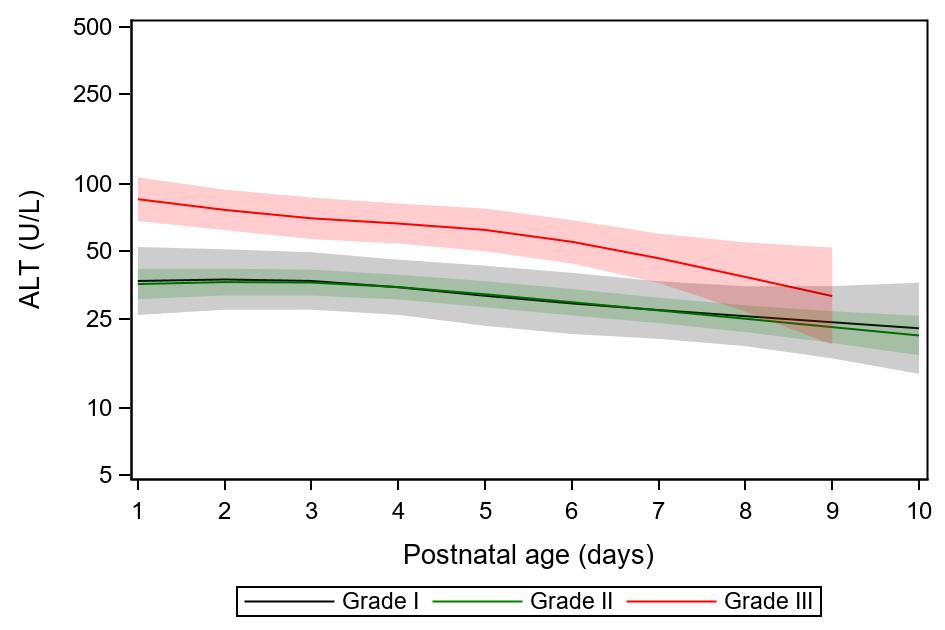


| ***4. AST*** |
| --- |

| ***4.1 Evolution all subjects*** |
| --- |

| ***Individual profiles*** |
| --- |


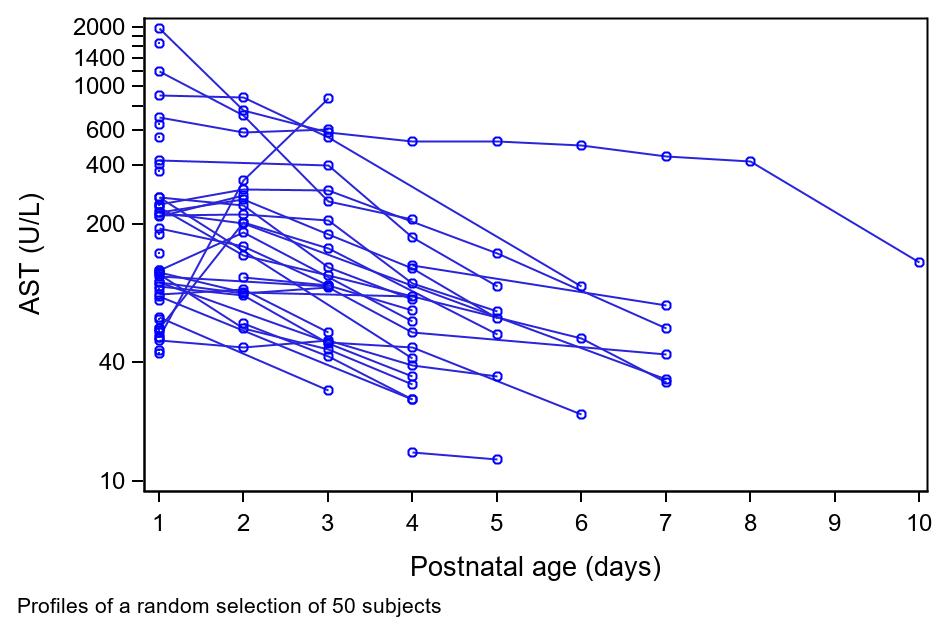


| ***Observed information*** |
| --- |

| *Analysis Variable : AST AST (U/L)* | | | | | | | | | | |
| --- | --- | --- | --- | --- | --- | --- | --- | --- | --- | --- |
| *Postnatal age (days)* | *N Obs* | *Mean* | *Std Dev* | *Median* | *Lower Quartile* | *Upper Quartile* | *Minimum* | *Maximum* | *90th Pctl* | *95th Pctl* |
| 1 | 270 | 349.00 | 573.65 | 154.50 | 79.00 | 345.00 | 5.00 | 4728.00 | 754.05 | 1320.50 |
| 2 | 154 | 285.62 | 468.39 | 141.00 | 72.00 | 282.00 | 4.00 | 3282.00 | 586.00 | 1038.00 |
| 3 | 142 | 218.57 | 357.54 | 96.50 | 55.00 | 191.00 | 14.00 | 2540.00 | 529.00 | 977.00 |
| 4 | 112 | 127.56 | 173.21 | 68.50 | 45.00 | 141.00 | 12.00 | 1128.00 | 295.00 | 484.00 |
| 5 | 68 | 101.50 | 141.66 | 64.50 | 43.00 | 88.00 | 9.00 | 997.00 | 273.00 | 321.00 |
| 6 | 44 | 113.45 | 165.64 | 54.50 | 34.50 | 112.50 | 9.00 | 763.00 | 223.00 | 502.00 |
| 7 | 30 | 68.20 | 79.70 | 43.50 | 31.00 | 75.00 | 7.00 | 446.00 | 128.00 | 169.00 |
| 8 | 12 | 67.33 | 114.32 | 28.50 | 21.00 | 44.00 | 5.00 | 417.00 | 125.00 | 417.00 |
| 9 | 9 | 46.33 | 28.12 | 35.00 | 27.00 | 51.00 | 21.00 | 103.00 | 103.00 | 103.00 |
| 10 | 7 | 40.43 | 39.53 | 26.00 | 18.00 | 33.00 | 18.00 | 129.00 | 129.00 | 129.00 |


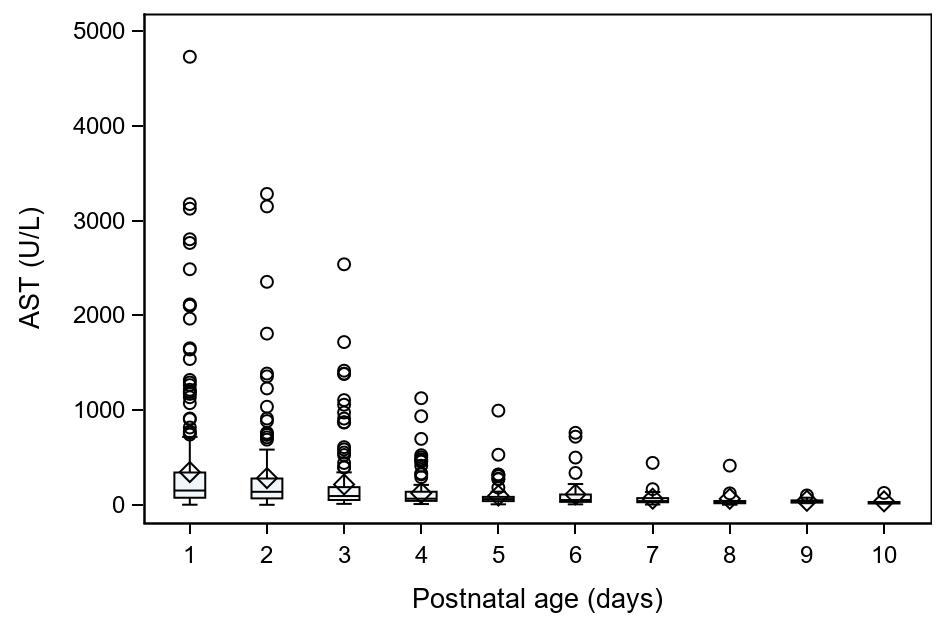


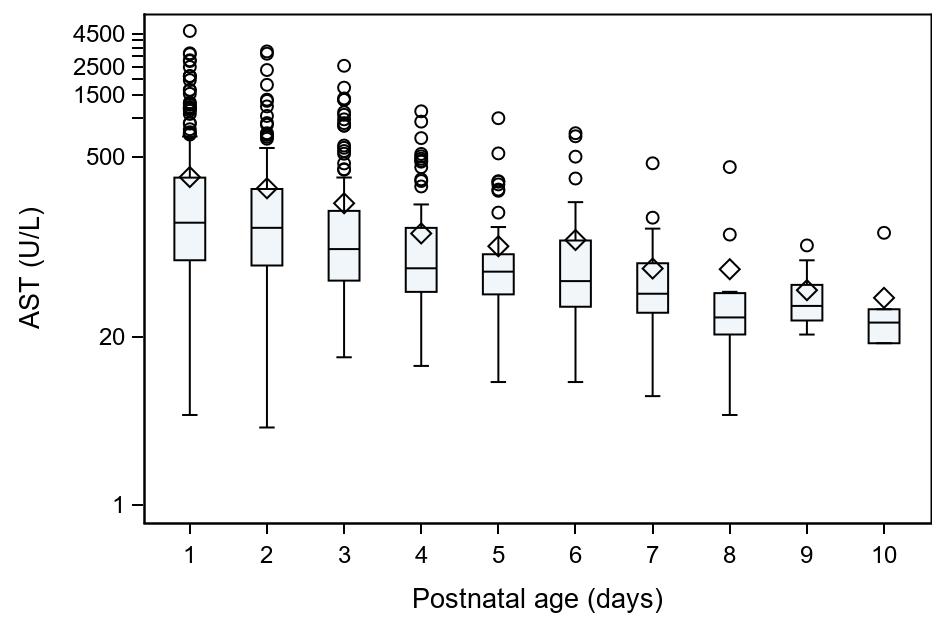


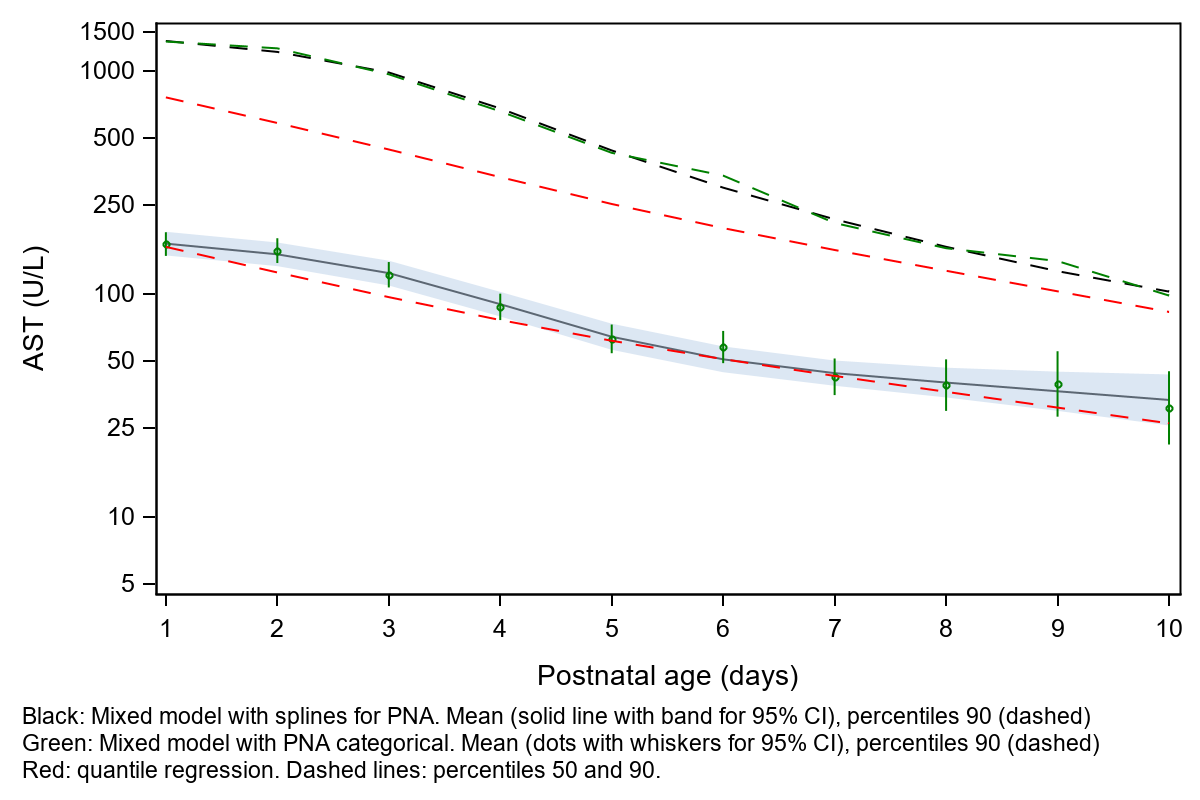


| ***4.2 As a function of HIE grade*** |
| --- |

| *label* | *Num DF* | *Den DF* | *F Value* | *P-value* |
| --- | --- | --- | --- | --- |
| Main effect HIE grade? | 2 | 236 | 20.66 | <.0001 |
| Interaction with HIE grade? | 6 | 351 | 2.69 | 0.0144 |


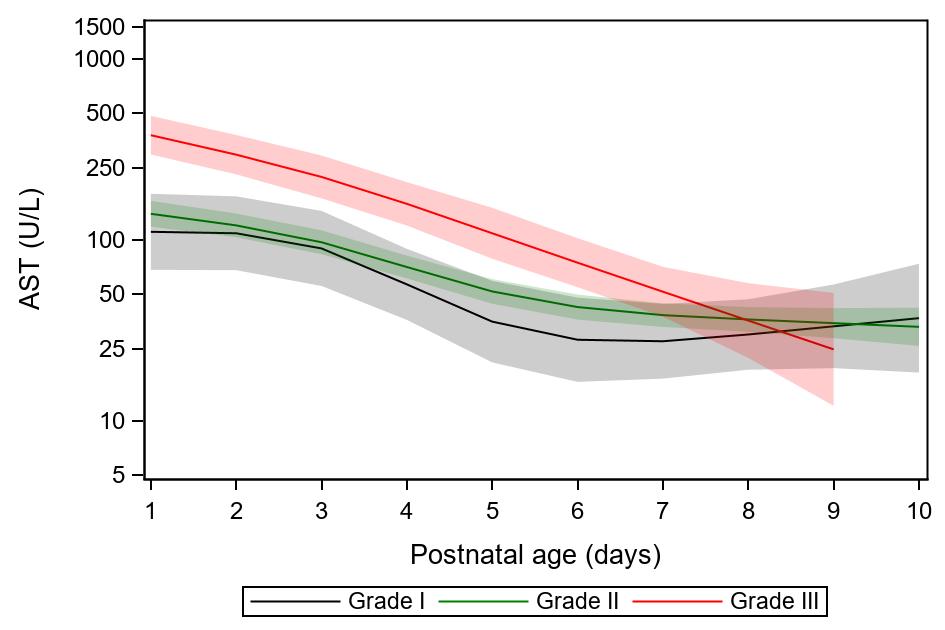


| ***5. De Ritis*** |
| --- |

| ***5.1 Evolution all subjects*** |
| --- |

| ***Individual profiles*** |
| --- |


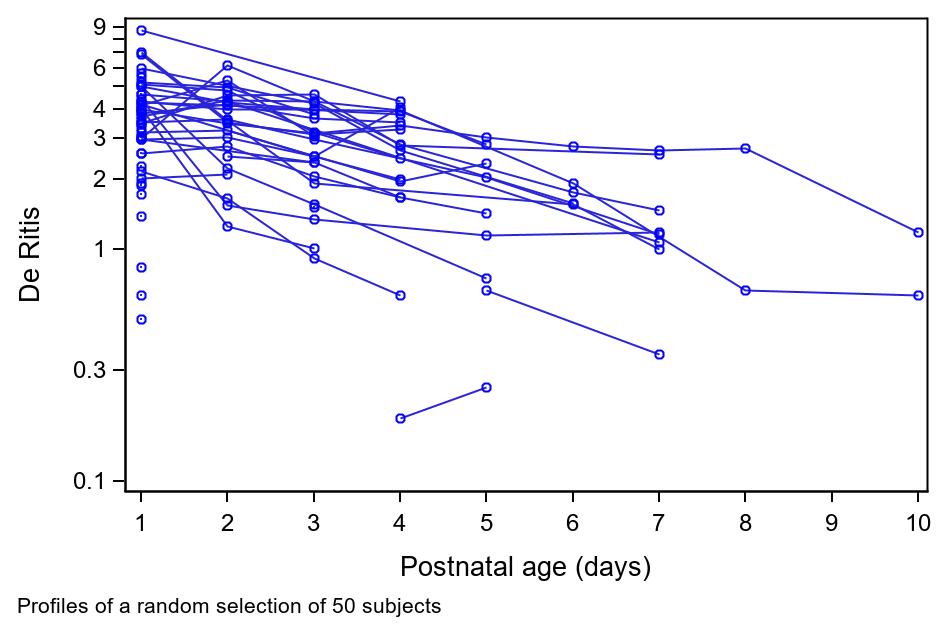


| ***Observed information*** |
| --- |

| *Analysis Variable : RITIS De Ritis* | | | | | | | | |
| --- | --- | --- | --- | --- | --- | --- | --- | --- |
| *Postnatal age (days)* | *N Obs* | *Median* | *Lower Quartile* | *Upper Quartile* | *Minimum* | *Maximum* | *90th Pctl* | *95th Pctl* |
| 1 | 237 | 3.47 | 2.58 | 4.68 | 0.11 | 13.31 | 6.45 | 7.30 |
| 2 | 127 | 3.25 | 2.40 | 4.20 | 0.13 | 9.15 | 5.15 | 5.67 |
| 3 | 116 | 2.70 | 1.96 | 3.56 | 0.22 | 12.71 | 4.33 | 5.22 |
| 4 | 94 | 2.24 | 1.35 | 3.25 | 0.19 | 7.83 | 4.33 | 5.54 |
| 5 | 55 | 1.68 | 1.05 | 2.35 | 0.25 | 4.21 | 2.96 | 3.91 |
| 6 | 33 | 1.56 | 1.06 | 1.88 | 0.27 | 4.40 | 2.77 | 3.00 |
| 7 | 24 | 1.12 | 0.83 | 1.44 | 0.15 | 5.00 | 2.55 | 2.67 |
| 8 | 12 | 1.39 | 0.49 | 1.78 | 0.23 | 2.73 | 2.30 | 2.73 |
| 9 | 9 | 1.91 | 1.20 | 2.26 | 0.51 | 2.86 | 2.86 | 2.86 |
| 10 | 7 | 1.20 | 1.00 | 1.65 | 0.63 | 2.54 | 2.54 | 2.54 |


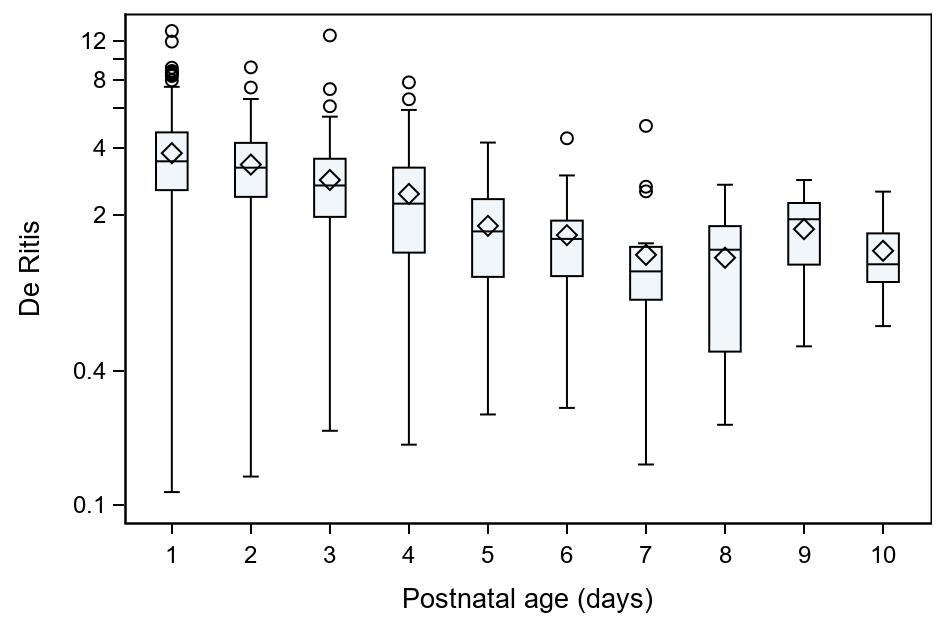


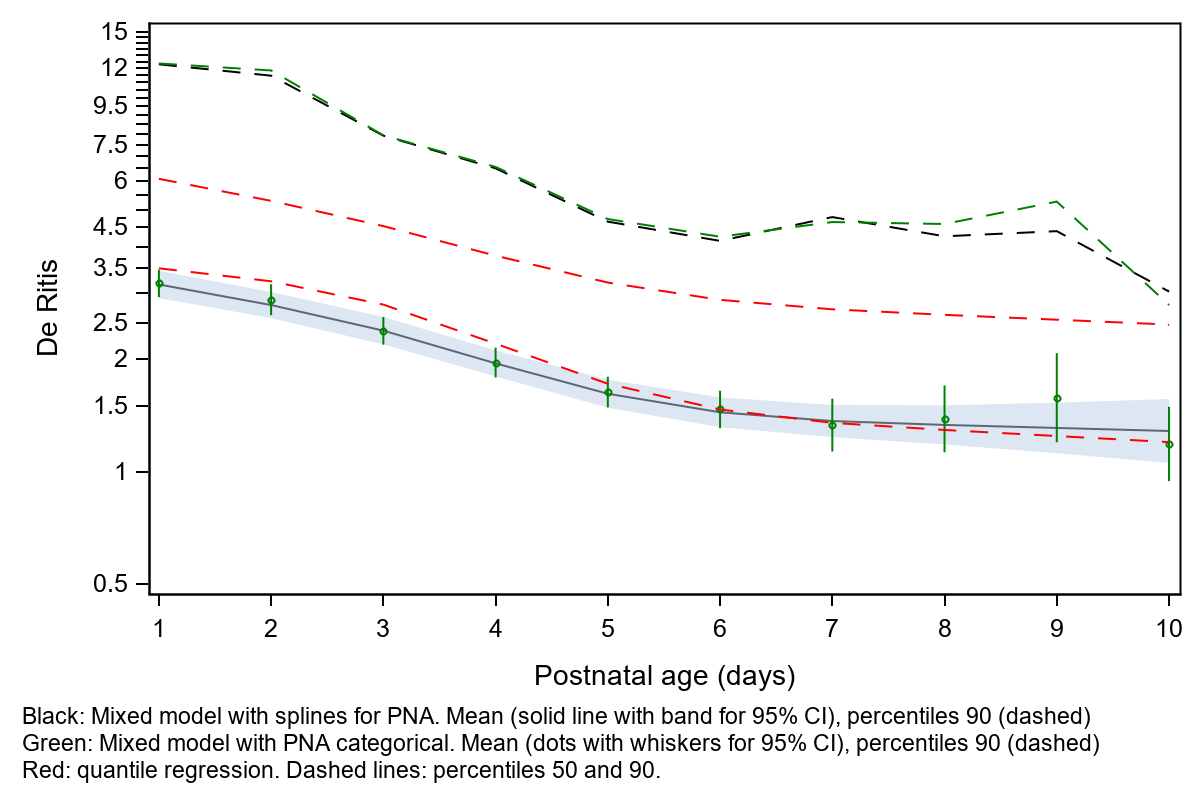


| ***5.2 As a function of HIE grade*** |
| --- |

| *label* | *Num DF* | *Den DF* | *F Value* | *P-value* |
| --- | --- | --- | --- | --- |
| Main effect HIE grade? | 2 | 203 | 0.63 | 0.5342 |
| Interaction with HIE grade? | 6 | 251 | 0.43 | 0.8611 |


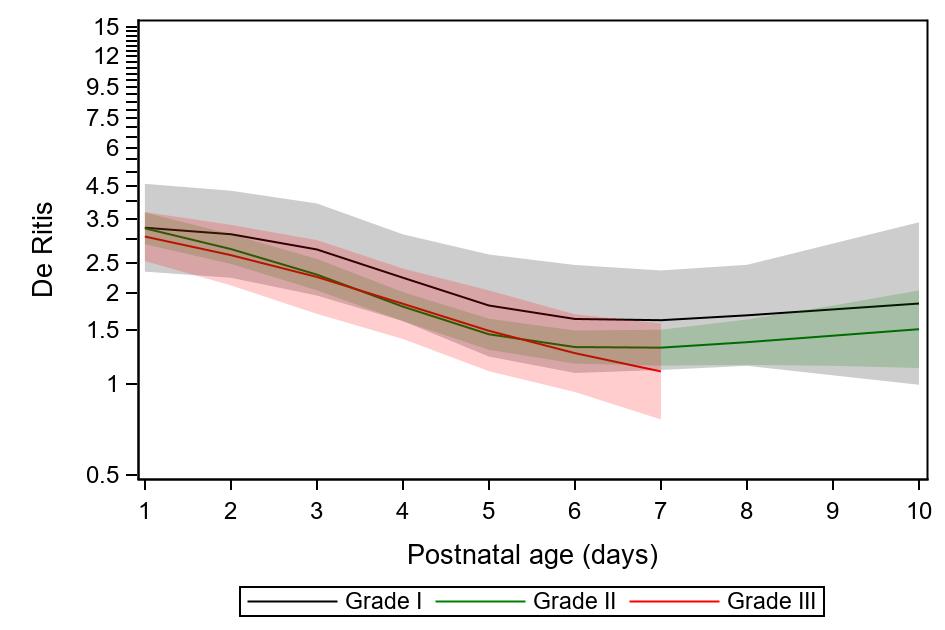

Supplement: Supplementary file 1 — Supplementary file1 (DOCX 1131 KB) [file 431_2026_7167_MOESM1_ESM.docx]
