# Supplementary material for: Time-dependent pattern of liver injury biomarkers in neonates with hypoxic-ischemic encephalopathy undergoing therapeutic hypothermia
Source: Eur J Pediatr. 2026 Jun 15;185(7):501. doi: 10.1007/s00431-026-07167-z (PMC13269461; doi:10.1007/s00431-026-07167-z)

**Supplemental materials**

**Search strategy, applied to PubMed (March 2022)**

("Hypothermia, Induced"[Mesh]) AND "Infant, Newborn"[Mesh]; OR therapeutic hypothermia AND neonate AND biochemistry’; OR ("Asphyxia Neonatorum"[Mesh]) AND "Blood Physiological Phenomena"[Mesh]; OR ("Hypothermia, Induced"[Mesh]) AND "Hypoxia-Ischemia, Brain"[Mesh]; OR (("Infant, Newborn"[Mesh]) AND "Hypothermia, Induced"[Mesh]) AND "Asphyxia Neonatorum"[Mesh]; OR Therapeutic hypothermia AND neonate AND bilirubin; OR Therapeutic hypothermia AND neonate AND liver function; OR Therapeutic hypothermia AND neonate AND ALT; OR Therapeutic hypothermia AND neonate AND AST; OR ("Asphyxia Neonatorum"[Mesh]) AND "Hypothermia, Induced"[Mesh]; OR ((therapeutic hypothermia) AND (neonate)) AND (glycemia); OR ((therapeutic hypothermia) AND (neonate)) AND (hypoglycemia); OR ((therapeutic hypothermia) AND (neonate)) AND (hyperglycemia); OR ((therapeutic hypothermia) AND (neonate)) AND (potassium); OR ((therapeutic hypothermia) AND (neonate)) AND (sodium); OR ((therapeutic hypothermia) AND (neonate)) AND (bicarbonate); OR ((therapeutic hypothermia) AND (neonate)) AND (liver).

**HepaCool statistical analysis, document added.**

**Supplementary Table S1.** Observed statistics of total bilirubin concentrations (mg/dL) over postnatal age, based on the pooled data.

| ***PNA (days)*** | ***N Obs*** | ***Median*** | ***Lower Quartile*** | ***Upper Quartile*** | ***Minimum*** | ***Maximum*** | ***90th*** |
| --- | --- | --- | --- | --- | --- | --- | --- |
| **1** | **270** | 2.7 | 1.8 | 3.6 | 0.6 | 7.4 | 4.8 |
| **2** | **247** | 3.9 | 2.5 | 5 | 0.1 | 8.1 | 6.3 |
| **3** | **218** | 4.5 | 3.1 | 6.2 | 0.4 | 10.8 | 6.8 |
| **4** | **186** | 4.5 | 2.5 | 6.3 | 0.4 | 11.3 | 7.4 |
| **5** | **145** | 3.6 | 1.9 | 5.3 | 0.4 | 12.6 | 8 |
| **6** | **105** | 3.4 | 1.4 | 5.4 | 0.1 | 19.9 | 7.3 |
| **7** | **74** | 3 | 1.6 | 4.9 | 0.5 | 15.7 | 6.9 |
| **8** | **22** | 4.9 | 2.1 | 9.5 | 0.6 | 16.5 | 12.1 |
| **9** | **15** | 2.9 | 0.9 | 7.4 | 0.4 | 12.9 | 11.4 |
| **10** | **9** | 2.2 | 1 | 2.7 | 0.8 | 3.5 | 3.5 |

**Supplementary Table S2.** Median, and lower and higher quartile over postnatal age for alanine aminotransferase (ALT) and aspartate aminotransferase (AST) concentrations (U/L) in term neonates, extracted from the International Neonatal Consortium neonatal lab value tool [19].

| **Day** | **ALT** | **AST** |
| --- | --- | --- |
| **1** | 27 (21-35) | 94 (72-133) |
| **2** | 26 (21-35) | 88 (68-121) |
| **3** | 26 (21-35) | 83 (65-112) |
| **4** | 25 (20-34) | 77 (61-103) |
| **5** | 24 (19-32) | 72 (57-95) |
| **6** | 23 (18-31) | 67 (53-89) |
| **7** | 23 (18-30) | 64 (51-85) |
| **8** | 22 (18-30) | 63 (50-83) |
| **9** | 22 (17-30) | 65 (51-86) |
| **10** | 22 (18-30) | 67 (53-88) |

**Supplementary Figure S1.** Total bilirubin concentrations over postnatal age (Figure S1a), and estimated mean (with 95% confidence interval) for total bilirubin (TB) over postnatal age (PNA, day 1 to day 10) according to hypoxic-ischemic encephalopathy severity grade (Figure S1b).

**Figure S1a**

**
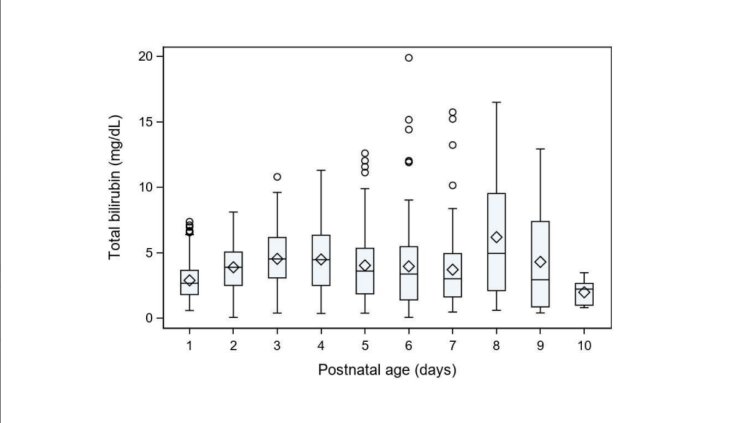

Figure S1b**


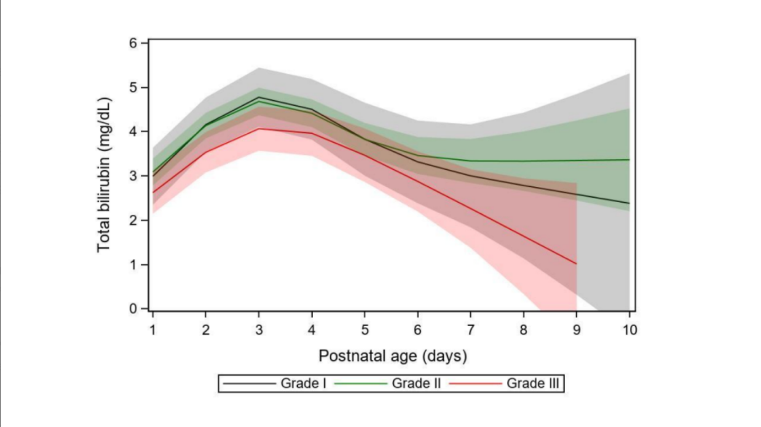

Supplement: Supplementary file 2 — Supplementary file2 (DOCX 182 KB) [file 431_2026_7167_MOESM2_ESM.docx]
